# Ultrasound Controlled‐Release Hydrogel Promotes Diabetic Wound Healing via Neuroimmune Modulation and Synergistic ROS Scavenging

**DOI:** 10.1002/advs.202516882

**Published:** 2026-01-21

**Authors:** Mofan Li, Mengxin Wang, Haonan Wang, Yang Sun, Yongyue Zhang, Shuyu Xu, Tianjiao Zhang, Shiti Shama, Xiaolong Liang, Shumin Wang

**Affiliations:** ^1^ Department of Ultrasound Peking University Third Hospital Beijing 100191 China

**Keywords:** calcitonin gene‐related peptide, diabetic wound, neuroimmune modulation, ultrasound‐responsive

## Abstract

Diabetic wound (DW) is a diabetes complication characterized by high morbidity and disability rates. Previous therapeutic systems focused on macrophages while neglecting the upstream regulatory factor of neuropeptide‐mediated neuroimmune communication. In addition, precise delivery is directly important for the treatment of DW. This study constructed an amphiphilic prodrug molecule MC by covalently conjugating calcitonin gene‐related peptide (CGRP) with manganese porphyrin (MnP). MC was then co‐assembled with DSPE‐PEG‐folic acid to form targeted nanoparticles MCF. Subsequently, MCF was loaded into an ultrasound‐responsive hydrogel to obtain the MCF@CA system, integrating neuroimmune modulation and reactive oxygen species (ROS) scavenging functions. Upon local administration, ultrasound triggering enables the on‐demand release of the nanodrug MCF from MCF@CA. Subsequently, FA targets M1 macrophages, prolonging wound retention time. MnP scavenges ROS, improving fibroblast function and promoting macrophage polarization towards an anti‐inflammatory phenotype. This study presents an ultrasound‐responsive hydrogel MCF@CA delivering targeted nanoparticles where CGRP regulates the regenerative transition of the immune microenvironment. Animal experiments confirmed that MCF@CA combined with ultrasound significantly promotes DW healing by enhancing collagen deposition, immune modulation, and improving blood supply. Therefore, this study provides an on‐demand controlled delivery platform with clear translational potential for diabetic wound therapy.

## Graphical abstract

This study presents an ultrasound‐responsive hydrogel (MCF@CA) that co‐delivers a neuropeptide (CGRP) and a ROS‐scavenging manganese porphyrin to diabetic wounds. The system restores neuro‐immune communication, reprograms macrophages toward an anti‐inflammatory phenotype, and clears excess ROS, thereby accelerating wound closure and promoting mature vessel formation in diabetic mice.

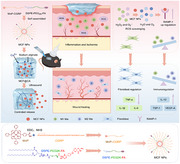

## 1 Introduction

Diabetic wound (DW) is a common complication of diabetes that can lead to infection, amputation, and increased patient mortality, posing a global public health challenge [1]. Wound healing is a dynamic process involving three phases: [2] inflammation, proliferation, and remodeling [3]. This process requires close coordination among various elements, including nerves, blood vessels, immune cells, and tissue cells [4]. However, in DW, hyperglycemia and secondary issues like insufficient blood supply and infection place the wound in a complex pathophysiological state, including excessive reactive oxygen species (ROS) accumulation, peripheral neuropathy, microcirculatory impairment, immune dysregulation, and fibroblast dysfunction [5, 6].

The immune system, particularly macrophages, plays a key role in tissue repair [7, 8]. Previously developed biomedical materials often focused on direct macrophage intervention [9], but such treatments have shown limited efficacy, and the clinical challenge of impaired DW healing remains severe [1]. Increasing evidence suggests that the numerous detrimental factors in DW are not independent but exhibit complex interactions [3, 7]. A major deficiency in current wound therapies is directly targeting macrophages while neglecting abnormalities in their upstream regulators. Diabetic peripheral neuropathy (DPN) is considered the most significant risk factor for DW [10]. Recent studies [11, 12, 13, 14, 15] have revealed the role of peripheral nerves in wound repair, demonstrating their crucial immunomodulatory role via neuropeptides. A recent study published in NATURE [12] uncovered the impact of calcitonin gene‐related peptide (CGRP)‐mediated neuroimmune communication on wound healing. During healing, CGRP promotes the transition from the inflammatory phase to the anti‐inflammatory and repair phase by modulating macrophage phenotype and upregulating the key downstream effector molecule thrombospondin‐1 (TSP‐1). However, in DW, sensory nerve damage reduces CGRP secretion, weakening its immunomodulatory effect on macrophages and trapping the wound in a prolonged inflammatory phase [12]. Concurrently, the oxidative stress environment in DW downregulates receptor activity modifying protein 1 (RAMP‐1), a critical co‐receptor regulating CGRP function on macrophage membranes [16, 17], further impairing this neuro‐immune interaction.

Therefore, an ideal therapeutic strategy should account for the influence of peripheral nerves—the upstream regulators—on wound immunity. Some studies have adopted strategies promoting peripheral nerve regeneration to restore neuronal regulation in DW healing [5]. However, the pathogenesis of DPN is complex, involving processes like axonal atrophy, Schwann cell demyelination, and reduced nerve regeneration. With its molecular mechanisms still unclear [18], no reliable therapy for DPN has yet received FDA approval [10]. Current treatments can only delay the progression of peripheral neuropathy and have limited efficacy for existing lesions [10, 19]. Therefore, employing a neural replacement strategy by directly delivering CGRP, which is deficient due to neuropathy, represents a more efficient therapeutic method. However, constrained by the high‐protease environment of DW and the short half‐life of peptide drugs, free CGRP degrades rapidly upon entering the wound. Furthermore, the immunomodulatory effect of CGRP relies on low‐dose, long‐term action [12]. Consequently, methods using direct delivery of free CGRP in previous studies struggled to achieve ideal results [12, 20, 21]. In summary, a system capable of controlling the drug release rate is needed to allow manual adjustment of drug release according to clinical therapeutic needs, solving the problem caused by rapid CGRP degradation. Ultrasound, with its non‐invasiveness, strong penetration, and high controllability, is an ideal drug release trigger [22, 23, 24, 25]. Calcium‐crosslinked sodium alginate hydrogel degrades under ultrasound [23, 26]. Compared to passive diffusion or environmentally responsive release hydrogel systems, this type of hydrogel offers superior controllability [27], allowing tailored release strategies for diverse clinical needs, making it an ideal universal platform for controlled drug release.

Besides immune dysregulation, oxidative stress is another major factor hindering DW healing. Excessive ROS cause dysfunction in components like macrophages, fibroblasts, and vascular endothelial cells, impeding processes such as collagen deposition, neovascularization, and re‐epithelialization [28]. On the other hand, ROS induce downregulation of RAMP‐1, the receptor for CGRP [16], inhibiting the regulatory role of neuroimmune communication. Therefore, scavenging ROS is an indispensable approach in DW treatment. Manganese porphyrin (MnP) possesses excellent catalase (CAT)‐like and superoxide dismutase (SOD)‐like activity [29, 30], but its hydrophobic nature limits its application in wound therapy. Covalently conjugating CGRP with MnP forms an amphiphilic molecule enabling the construction of self‐assembling nanoparticles, improving MnP dispersion in aqueous solutions.

Additionally, research in wound therapy often employs local delivery systems, which avoid drug accumulation in non‐target organs. Therefore, these studies rarely utilize active targeting strategies. However, in DW, non‐targeted drug delivery systems may still face off‐target issues [31]. Besides macrophages, various cells including fibroblasts express CGRP receptors [17], and the biological effects of CGRP on these cells are not fully understood; non‐targeted delivery strategies may introduce uncertainty into treatment. Leveraging the high expression of folate receptors on pro‐inflammatory M1 macrophages [32], folic acid (FA) targeting enhances drug delivery efficiency and avoids potential off‐target effects.

**FIGURE 1:**
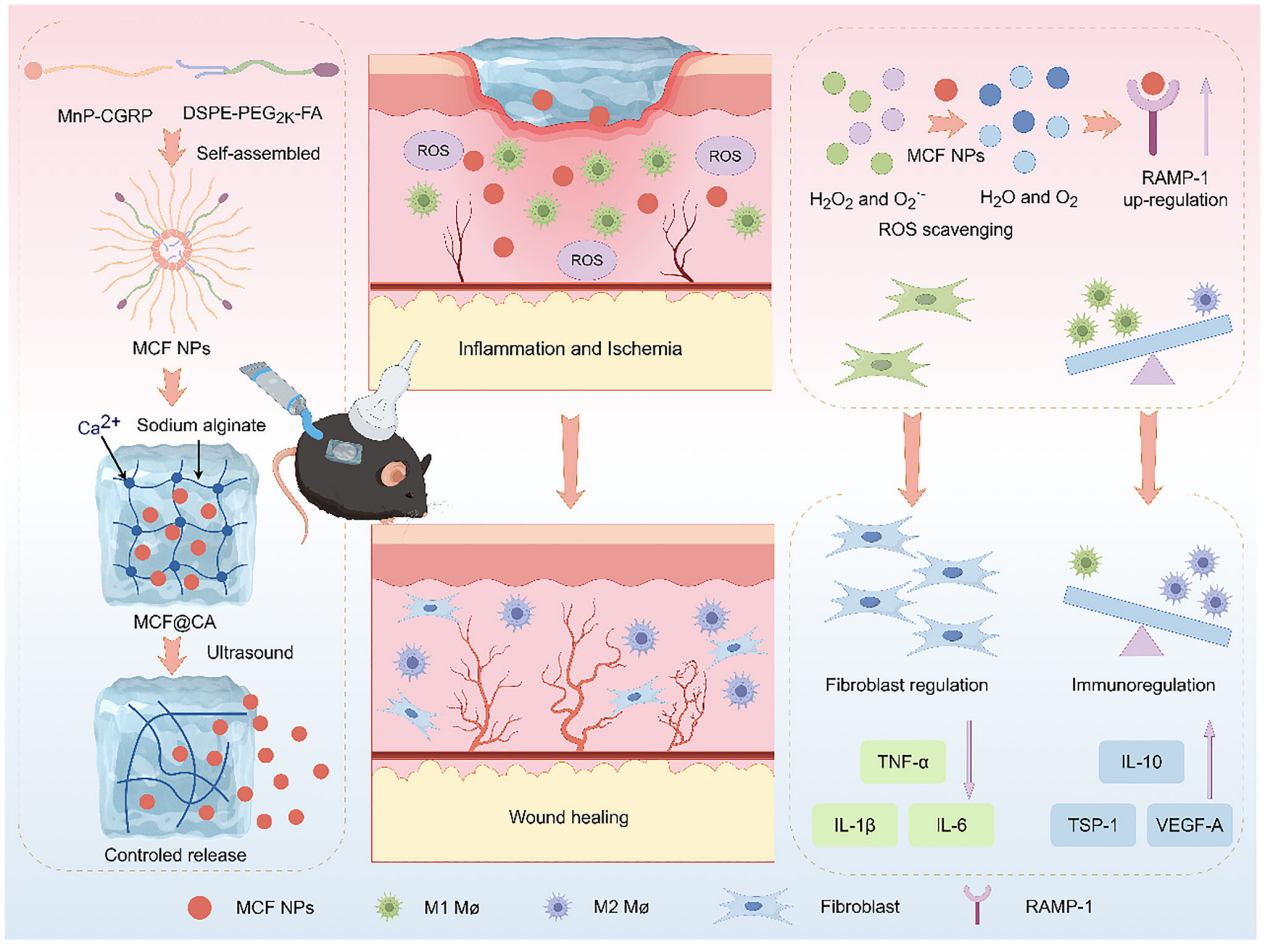
Schematic of the structural units and fabrication process of MCF@CA, and the mechanism of the treatment for diabetic wounds. The picture was created by Figdraw.

In summary, while recent advances have demonstrated the potential of CGRP delivery for immunomodulation and ROS‐scavenging materials for alleviating oxidative stress in diabetic wounds, these approaches often target only a single aspect of the complex pathology, thereby limiting their therapeutic efficacy. Particularly, strategies relying on the direct application of free CGRP peptides are constrained by poor stability and a lack of targeted delivery, which can compromise local efficacy and raise safety concerns. A comprehensive strategy capable of simultaneously achieving targeted delivery of the neuropeptide CGRP, integrating synergistic ROS‐scavenging capability, and enabling controlled drug release remains unexplored. Such an integrated approach is crucial for breaking the vicious cycle of chronic inflammation and impaired healing. To this end, this study aims to construct a synergistic therapeutic platform that integrates the aforementioned multifunctional capabilities into a single, targeted system designed to enhance local action and minimize systemic effects. We designed a controllable‐release drug delivery system (Figure 1). MnP, possessing ROS scavenging ability, was covalently conjugated via amidation reaction with CGRP, possessing immunomodulatory ability, to form an amphiphilic molecule (MC). MC was then co‐assembled with FA‐modified DSPE‐PEG to form targeted nanoparticles MCF (MCF NPs). MCF NPs were encapsulated within ultrasound‐controlled‐release calcium‐crosslinked sodium alginate hydrogel to construct the MCF@CA system, integrating neuroimmune modulation and ROS scavenging capabilities for promoting DW healing. In in vitro experiments and treatment studies using a diabetic mouse wound model, MCF@CA demonstrated excellent ROS scavenging, immunomodulation, fibroblast functional improvement, and pro‐angiogenic capabilities, significantly accelerating DW healing.

## 2 Results and Discussion

### 2.1 Synthesis and Characterization of MCF@CA

**FIGURE 2:**
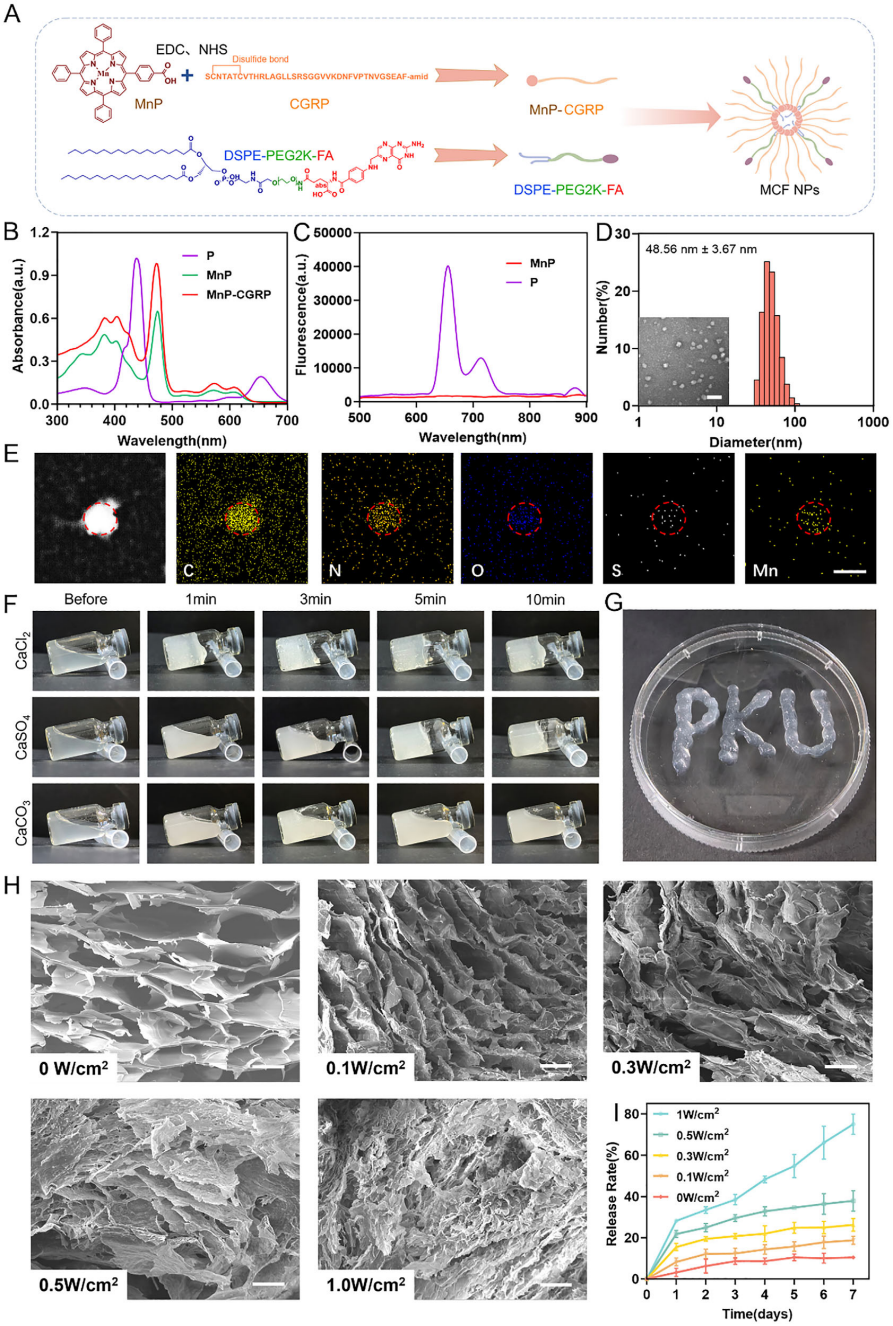
Preparation and characterization of MCF NPs and MCF@CA. (A) Schematic illustration of MCF NPs preparation. The picture was created by Figdraw. (B) UV–vis absorption spectra of porphyrin, MnP, and MnP‐CGRP. (C) Fluorescence spectra of porphyrin and MnP. (D) TEM image of MCF NPs and their hydrodynamic size/zeta potential measured by dynamic light scattering. Scale bar: 100 nm. (E) EDS results of MCF NPs revealing the elemental distribution of C, N, O, S, and Mn atoms. Scale bar: 100 nm. (F) Gelation time of hydrogels formed by mixing sodium alginate with different calcium salts. (G) Hydrogel using CaSO_4_ as the Ca^2+^ donor exhibits injectability during the initial crosslinking phase. (H) SEM images of MCF@CA hydrogels after ultrasound irradiation at different acoustic powers (0, 0.1, 0.3, 0.5, 1.0 W/cm^2^). Scale bar: 100 nm. (I) Cumulative release rate of MCF NPs from hydrogels after ultrasound irradiation at different acoustic powers (n = 3).

To materialize the above therapeutic strategy, we synthesized the manganese porphyrin‐conjugated CGRP prodrug and subsequently fabricated the ultrasound‐responsive hydrogel system. First, 5‐(4‐Carboxyphenyl)‐10,15,20‐triphenylporphyrin and manganese chloride were reacted via chelation to synthesize MnP. UV–vis spectrophotometry and fluorometry showed a shift in the absorption peak from 438 nm (porphyrin) to 475 nm (MnP) (Figure 2B), and the intrinsic fluorescence of porphyrin disappeared (Figure 2C), confirming the reaction. Subsequently, MnP was covalently conjugated to CGRP via amidation reaction, forming the amphiphilic molecule MnP‐CGRP (MC) (Figure 2A). Mass spectrometry revealed molecular weight changes before and after the reaction, confirming successful conjugation (Figure S1A,B). MC and DSPE‐PEG2K‐FA were prepared into self‐assembling nanoparticles MCF using the ethanol injection method. The average hydrodynamic diameter of MCF NPs measured by dynamic light scattering was 48.56 ± 3.67 nm, with an average zeta potential of 32.62 ± 0.68 mV (Figure 2D), indicating good dispersion. Transmission electron microscopy (TEM) showed the particle size of MCF NPs was 23.50 ± 4.92 nm (Figure 2D; Figure S2). The larger size obtained from DLS measurement is attributed to the hydration layer and solvation effects in solution. Energy dispersive spectrometry (EDS) confirmed the presence of manganese and sulfur elements within the MCF NPs (Figure 2E).

To achieve sufficient crosslinking strength, we used 3% sodium alginate and 60 mM calcium salt as the crosslinking concentration [22, 26]. MCF NPs were dispersed in a pre‐dissolved sodium alginate solution, and calcium salt was added as a crosslinker to form the MCF@CA hydrogel. To achieve a suitable gelation time for wound application, we investigated the gelation time using CaCl_2_, CaSO_4_, and CaCO_3_, three calcium salts with different solubilities. Calcium sulfate suspension mixed with sodium alginate solution formed a hydrogel within five minutes (Figure 2F). This is due to the low solubility of calcium sulfate, allowing slow release of Ca^2^^+^ ions into the alginate solution. This property endows the hydrogel with injectability during the initial mixing phase of the calcium ion donor and alginate solution (Figure 2G). In contrast, calcium chloride formed a hydrogel immediately upon mixing due to high solubility, making it difficult to adapt to variable wound topography and preventing injection into deeper wound layers; while calcium carbonate failed to crosslink due to insolubility (Figure 2F). Most hydrogels reported previously release drugs passively via diffusion or environmental response [22], which is insufficient to meet the diverse and individualized clinical demands for drug dosage control. Ultrasound offers advantages of controllability and non‐invasiveness [33]. Regulating drug release speed by adjusting ultrasound power can satisfy different clinical needs for personalized treatment based on patient conditions [34]. Under ultrasound, the calcium‐dependent crosslinked network in the alginate hydrogel disintegrates, forming a porous structure on the hydrogel surface [22, 26], accelerating the release of encapsulated drugs. Scanning electron microscopy (SEM) revealed the surface morphology of the hydrogel after exposure to different ultrasound powers. As ultrasound power increased, the crosslinked network on the hydrogel surface was progressively disrupted (Figure 2H). Subsequently, we conducted drug release experiments to determine if MCF@CA could control the release rate of MCF NPs by changing ultrasound power. We used PBS to simulate tissue fluid and applied ultrasound for 3 min daily. The release rate of MCF NPs from the hydrogel increased with higher ultrasound power (Figure 2I), confirming the potential of MCF@CA hydrogel for adjustable drug release speed according to therapeutic needs.

### 2.2 Folic Acid Improves the Pharmacokinetics of MCF NPs

**FIGURE 3:**
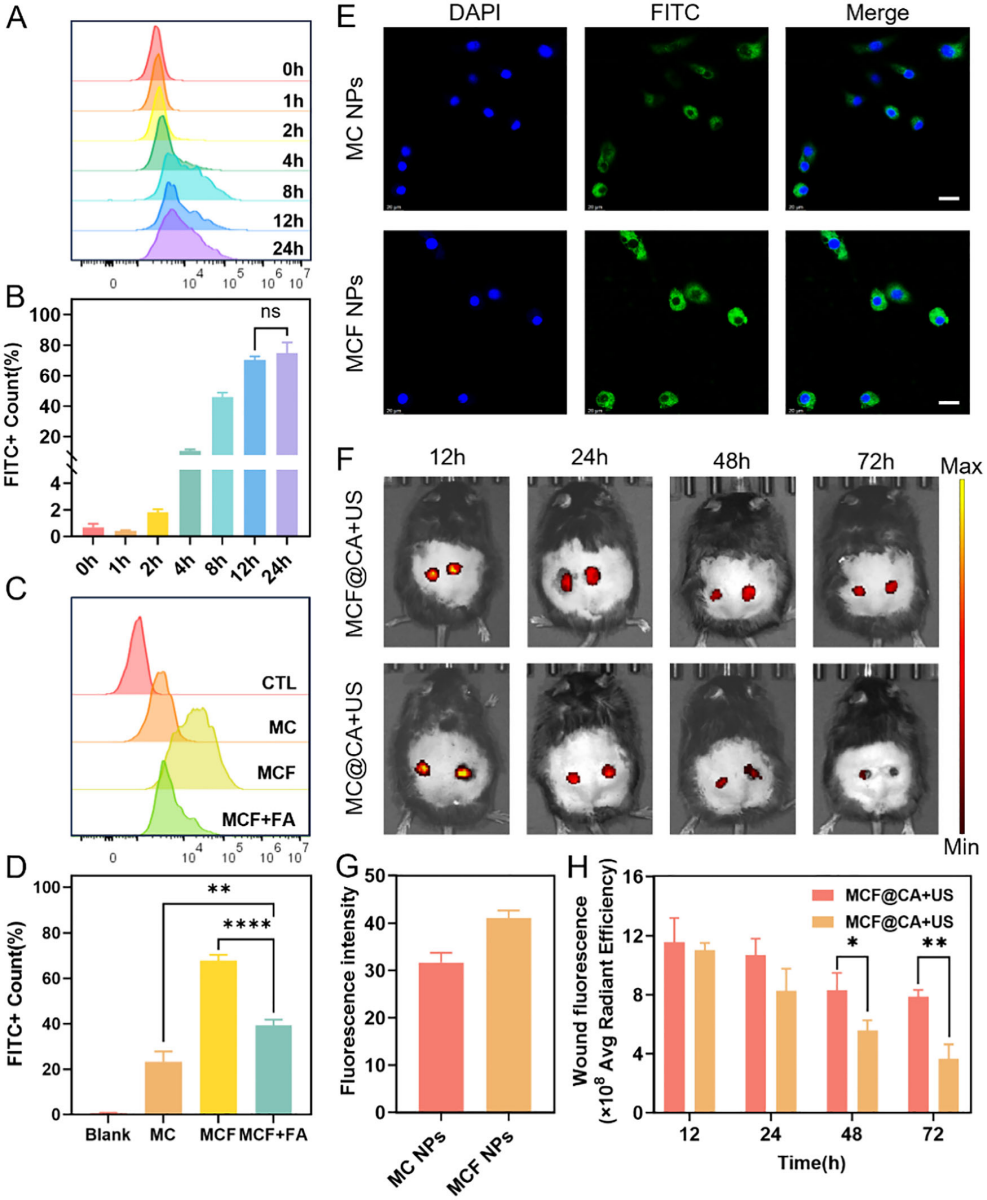
Uptake and Retention of MCF@CA. (A) Time‐dependent uptake of MCF NPs by RAW 264.7 cells analyzed by flow cytometry. (B) Quantitative analysis of FITC^+^ cell percentage over time in RAW 264.7 cells incubated with MCF NPs (n = 3). Statistical significance was determined by one‐way ANOVA. (C) Flow cytometry comparison of RAW 264.7 cell uptake after 12 h incubation with non‐targeted nanoparticles (MC NPs), MCF NPs, and folate receptor‐blocked MCF NPs. (D) Quantification of FITC^+^ cell percentage after 12 h incubation with MC NPs, MCF NPs, and folate‐blocked MCF NPs (n = 3). Statistical significance was determined by one‐way ANOVA. (E) Confocal microscopy images comparing intracellular uptake of MC NPs and MCF NPs by RAW 264.7 cells at 12 h, with quantitative fluorescence intensity analysis (G) (n = 3). Scale bar: 20 µm. (F) IVIS imaging comparison of retention kinetics between MCF NPs and non‐targeted nanoparticles in diabetic mouse wounds, with quantitative fluorescence intensity analysis (H) (n = 3). Statistical significance was determined by Student's *t*‐test. *p < 0.05, **p < 0.01, ***p < 0.001, ****p < 0.0001.

Next, we used LPS‐polarized RAW 264.7 cells as a model to investigate M1 macrophage uptake of nanoparticles, specifically the effect of the targeting molecule folic acid (FA). To track MCF NPs intracellularly, we labeled them with FITC. Flow cytometry showed that M1 macrophage uptake of MCF NPs increased rapidly within 4 h of incubation and reached saturation at 12 h (Figure 3A,B). Conversely, uptake was significantly reduced when DSPE‐PEG2K was used instead of DSPE‐PEG2K‐FA in the NPs (Figure 3C,D). To confirm FA's role in enhancing uptake, we pre‐treated macrophages with high concentrations of free FA to block folate receptors. Flow cytometry confirmed that free FA pre‐treatment also reduced uptake (Figure 3C,D). Confocal microscopy further validated these results. Compared to the group without FA, cells incubated with NPs containing the targeting molecule DSPE‐PEG2K‐FA showed higher intracellular fluorescence intensity after 12 h (Figure 3E,G), indicating greater nanoparticle accumulation. These findings demonstrate that FA enhances M1 macrophage uptake of MCF NPs.

Studies indicate that the immunomodulatory efficacy of CGRP in DW relies on sustained action rather than short‐term, high‐dose delivery [12]. Therefore, we used Lepr^db/db^ mice to investigate whether MCF NPs released from the hydrogel could remain in the wound long enough. To track retained MCF NPs, we labeled them with Cy5.5 to monitor their accumulation in the wound via fluorescence intensity. Twelve hours after applying MCF@CA to the wound, the surface hydrogel was removed, and an in vivo imaging system (IVIS) was used to track retained nanoparticles. At 12 h post‐administration, MCF NP retention showed no significant difference compared to non‐targeted NPs (without FA). However, at 48 h, fluorescence intensity in the group receiving NPs without FA decreased by half (Figure 3F,H). In contrast, MCF NPs with FA maintained high fluorescence intensity even at 72 h (Figure 3F,H). This evidence indicates that FA significantly increases the retention time of MCF NPs in DW, which is beneficial for enhancing CGRP's immunomodulatory effect. It also suggests that a single drug release could provide effective treatment for up to 3 days, potentially avoiding the need for frequent ultrasound application and dressing changes in clinical settings.

### 2.3 MCF@CA Scavenges ROS and Promotes Fibroblast Proliferation and Migration

**FIGURE 4:**
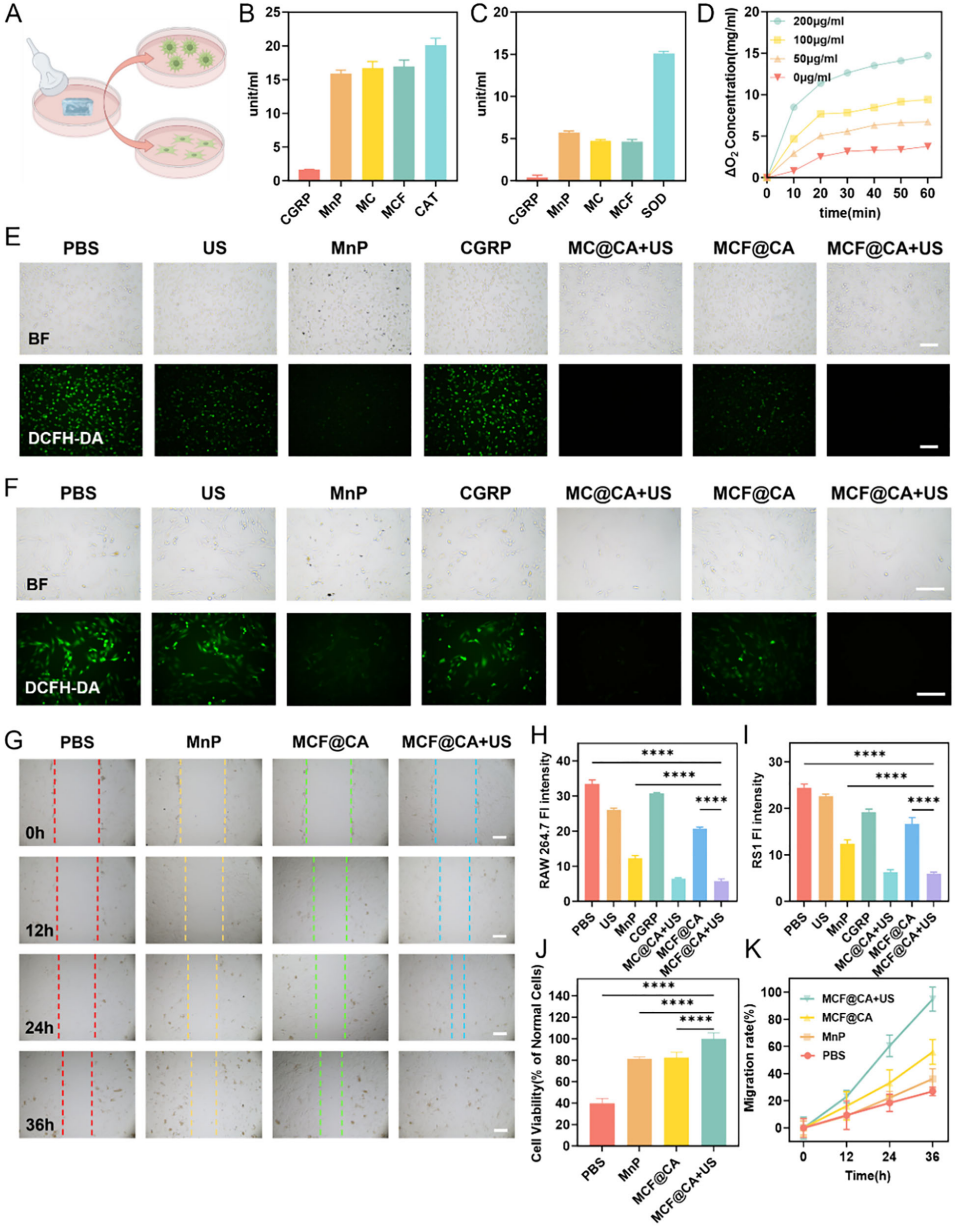
In Vitro Validation of ROS Scavenging Capacity of MCF@CA and its Effects on Fibroblasts. (A) Schematic diagram of cellular experiments. The picture was created by Figdraw. (B) Catalase‐like activity of MCF NPs measured using a catalase assay kit (n = 3). (C) Superoxide dismutase‐like activity of MCF NPs measured using a superoxide dismutase assay kit (n = 3). (D) Oxygen production from hydrogen peroxide decomposition by MCF NPs at different concentrations (0, 50, 100, 200 µg/mL) (n = 3). (E), (H) Fluorescence images of intracellular ROS in H_2_O_2_‐incubated RAW 264.7 cells under different treatment conditions (n = 3). Scale bar: 500 µm. (F, I) Fluorescence images of intracellular ROS in H_2_O_2_‐incubated RS1 cells under different treatment conditions (n = 3). Scale bar: 200 µm. (G, K) Scratch assay evaluating RS1 cell migration rate under different treatment conditions (n = 3). (J) Cell viability of H_2_O_2_‐incubated RS1 cells under different treatment conditions (relative to untreated RS1 cells as control, n = 3). Statistical significance was determined by one‐way ANOVA. *p < 0.05, **p < 0.01,***p < 0.001,****p < 0.0001.

Factors like high glucose‐induced cellular oxidative stress (Figure S3A,B) contribute to the inflammatory microenvironment [1, 35, 36], a major reason for impaired DW healing. Specifically, high glucose dysregulates multiple intracellular glucose metabolism pathways, leading to excessive production of superoxide anion (O_2_•^−^) and hydrogen peroxide (H_2_O_2_). These can further generate more damaging species like hydroxyl radicals (·OH) and singlet oxygen (^1^O_2_) under specific conditions [37]. Therefore, we incorporated MnP into MCF NPs, utilizing its SOD‐like and CAT‐like activity to scavenge intracellular ROS and reverse the cellular oxidative stress state [29]. In vitro experiments confirmed that MnP, even after covalent conjugation to CGRP and self‐assembly into NPs, retained functions similar to natural superoxide dismutase and catalase, capable of catalytically decomposing O_2_•^−^ and H_2_O_2_ (Figure 4B,C). Additionally, we examined the oxygen generation capacity of MCF NPs from H_2_O_2_ decomposition in vitro. Dissolved oxygen levels gradually increased with higher MCF NP concentrations and longer reaction times (Figure 4D), confirming good CAT‐like activity. The generated oxygen can ameliorate intracellular hypoxia (Figure 4; Figure S4A,B), which aids wound healing [5].

Subsequently, we established cellular models to verify MCF@CA hydrogel's ability to scavenge intracellular ROS. We confirmed that high glucose treatment induces cellular oxidative stress, and intracellular ROS levels gradually increase with rising glucose concentration in the medium (Figure S3A,B). However, H_2_O_2_ is a more reliable method for inducing oxidative stress than high glucose. Relevant studies predominantly use H_2_O_2_ (100 µM, 4 h) to simulate oxidative stress induced by high glucose [38, 39, 40]. Therefore, we used H_2_O_2_ to construct cellular models in subsequent experiments and assessed intracellular ROS levels using the ROS probe 2',7'‐dichlorofluorescin diacetate (DCFH‐DA). After H_2_O_2_ treatment, both macrophages (RAW 264.7) (Figure 4E,F) and fibroblasts (RS1) (Figure 4H,I) exhibited strong green fluorescence in PBS, indicating elevated intracellular ROS levels. Isolated ultrasound treatment or CGRP alone could not scavenge intracellular ROS. However, after 12 h of incubation with medium treated with MCF@CA (Figure 4A), ROS levels significantly decreased in both RAW 264.7 and RS1 cells. Notably, MCF@CA showed better ROS scavenging efficacy than free MnP. This is likely because hydrophobic free MnP spontaneously aggregates into particles via π‐π stacking [41], whereas in MCF@CA, MnP is covalently linked to CGRP and self‐assembled into NPs, improving the dispersion of MCF NPs in water and enhancing ROS scavenging efficiency.

Fibroblasts are crucial participants in wound closure and remodeling [42]. Oxidative stress‐induced fibroblast dysfunction is a key factor hindering DW healing [4, 43]. Having demonstrated MCF@CA's ability to scavenge ROS in fibroblasts, we further evaluated its effect on fibroblast proliferation and migration. First, using the CCK‐8 assay, we determined the impact of different H_2_O_2_ concentrations on fibroblast proliferation. RS1 cell proliferation decreased progressively with increasing H_2_O_2_ concentration, reaching ∼50% inhibition at 200 µM, while cells almost completely lost viability at 400 µM (Figure S5). Therefore, we selected 200 µM H_2_O_2_ to simulate oxidative stress in subsequent experiments. The CCK‐8 assay showed that RS1 cells treated with medium conditioned with free MnP or MCF@CA exhibited restored proliferation compared to PBS‐treated cells (Figure 4J). However, due to the poorer dispersion of free MnP compared to MCF NPs, its ROS scavenging ability was lower than that of MCF@CA. Simultaneously, since sodium alginate hydrogel degrades naturally at a slow rate, and ultrasound promotes its degradation, the MCF@CA + Ultrasound (US) group, which released more MCF NPs, demonstrated the best ROS scavenging ability compared to the group without ultrasound. This further corroborates the ability of MCF@CA to alter drug release speed under ultrasound control. Subsequently, we investigated via a scratch wound assay whether MCF@CA's ROS scavenging function affects fibroblast migration ability (Figure 4G,K). Fibroblasts treated with PBS migrated very slowly, with wound closure still below 40% after 36 h. Groups treated with free MnP or MCF@CA without ultrasound application showed some increase in migration speed. In contrast, fibroblasts treated with MCF@CA and ultrasound migrated faster, achieving complete wound closure within 36 h.

In summary, MCF@CA possesses SOD‐like and CAT‐like activities, endowing it with ROS scavenging capability to eliminate intracellular oxidative stress induced by high glucose. More importantly, MCF@CA improves fibroblast proliferation and migration by scavenging ROS, which is beneficial for DW healing.

### 2.4 MCF@CA Promotes Macrophage Phenotype Switching In Vitro

**FIGURE 5:**
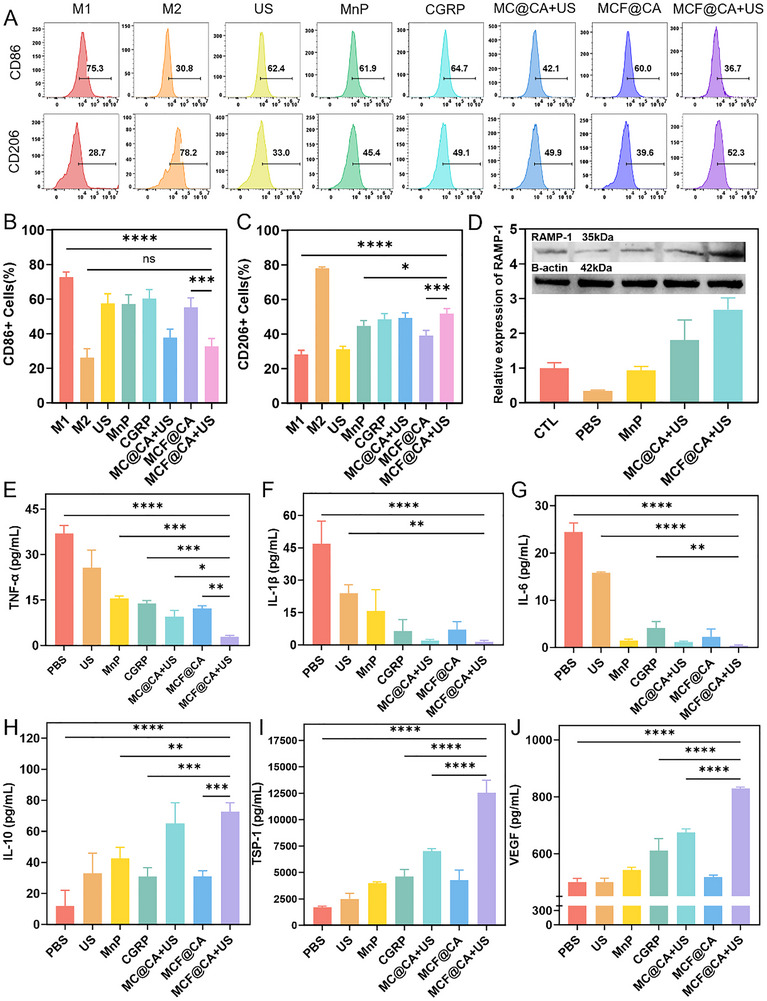
In Vitro Validation of Macrophage Phenotype Modulation by MCF@CA. (A) Flow cytometric analysis of M1 phenotype marker (CD86) and M2 phenotype marker (CD206) in macrophages under different treatment conditions. (B) Quantitative analysis of CD86^+^ cell proportion under different treatments (n = 3). Statistical significance was determined by one‐way ANOVA. (C) Quantitative analysis of CD206^+^ cell proportion under different treatments (n = 3). (D) Western Blot detection of RAMP‐1 expression in macrophages under different treatments (representative of three experiments). (E–J) ELISA detection of secretion levels for TNF‐α (E), IL‐1β (F), IL‐6 (G), IL‐10 (H), TSP‐1 (I), and VEGF (J) in macrophages under different treatments (n = 3). Statistical significance was determined by one‐way ANOVA. *p < 0.05, **p < 0.01,***p< 0.001,****p < 0.0001.

Macrophage phenotype switching is a key driver for transitioning wounds from the inflammatory phase to the repair phase during healing [4, 6]. However, in DW, this polarization process is disrupted by factors including the lack of upstream regulators, high glucose, poor perfusion, and oxidative stress, which collectively cause intracellular metabolic abnormalities. This leads to persistent inflammation in DW and impairs the pro‐healing functions of endothelial cells, keratinocytes, and fibroblasts [4, 37]. Therefore, promoting the polarization of M1 macrophages towards the M2 phenotype is an important therapeutic strategy for DW [6, 44]. We isolated mouse bone marrow‐derived macrophages (BMDMs), induced their differentiation into M1 macrophages using lipopolysaccharide (LPS), treated them with H_2_O_2_ to simulate oxidative stress, and used interleukin‐4 (IL‐4) to induce M2 polarization as a control, to investigate MCF@CA's modulatory effect on macrophages. Phenotypes were distinguished using CD86 (M1 marker) and CD206 (M2 marker) [5]. Flow cytometry results (Figures 5A‐C) showed that isolated ultrasound, free CGRP, or free MnP had weak modulatory effects on macrophage phenotype. Although the percentage of CD86+ cells decreased to some extent, it remained above 60%. Conversely, treatment with free CGRP or free MnP increased the percentage of CD206+ cells, but it remained below 50%. This shows that either CGRP delivery or ROS scavenging alone can modulate macrophages toward an anti‐inflammatory phenotype. Additionally, the hydrogel group without ultrasound, due to insufficient release of MCF NPs, was also inadequate for modulating macrophage phenotype. In contrast, compared to other groups, macrophages incubated with MCF@CA‐conditioned medium had a significantly reduced percentage of CD86+ M1 phenotype cells (Figure 5A,B) and a significantly increased percentage of CD206+ M2 phenotype cells (Figure 5A,C), indicating effective promotion of an anti‐inflammatory phenotype. It should be noted that the hydrogel with non‐targeted particles (MC@CA) and MCF@CA had similar regulatory effects on macrophages. This is because the phenotype modulation process requires continuous drug action on cells for 48 h, far exceeding the time for cellular uptake of MCF NPs to reach saturation (12 h). Therefore, intracellular nanoparticle concentration had reached an effective level regardless of targeting.

In the macrophage phenotype modulation experiment, we found that macrophages incubated with MCF@CA‐conditioned medium showed a lower M1 and higher M2 ratio than those treated with free CGRP or free MnP alone. We speculate that MnP's ROS scavenging function enhances CGRP's regulatory effect on macrophages. Specifically, previous studies indicate that the expression level of RAMP‐1, the co‐receptor for CGRP, regulates CGRP's biological effects [17]. Furthermore, oxidative stress downregulates RAMP‐1 expression [16]. Therefore, we hypothesized that MCF@CA scavenges ROS within macrophages, upregulating RAMP‐1 and thereby amplifying CGRP's biological effects. Western blot analysis validated this hypothesis. Compared to the untreated control group (CTL), H_2_O_2_ treatment significantly downregulated RAMP‐1 expression in macrophages. Conversely, free MnP, MC@CA, or MCF@CA upregulated RAMP‐1 expression, with MCF@CA showing the most significant effect (Figure 5D). Thus, we propose MCF@CA amplifies CGRP's biological effects by scavenging ROS and upregulating RAMP‐1 expression on macrophages.

Cytokines are important carriers for macrophages to exert immunomodulatory functions. Excessive secretion of pro‐inflammatory cytokines keeps the wound in a persistent inflammatory state, preventing progression to the proliferative phase; while anti‐inflammatory cytokines help regulate the wound immune microenvironment and promote healing. Therefore, we used enzyme‐linked immunosorbent assay (ELISA) to assess the impact of MCF@CA on the secretion of bioactive molecules by macrophages (Figure 5E‐J). After inducing macrophage M1 polarization with LPS and stimulating with H_2_O_2_ to simulate oxidative stress induced by high glucose, macrophages secreted large amounts of pro‐inflammatory cytokines TNF‐α, IL‐1β, and IL‐6, while barely expressing the anti‐inflammatory cytokine IL‐10. Ultrasound treatment alone partially reduced the secretion of pro‐inflammatory cytokines and increased the secretion of the anti‐inflammatory cytokine IL‐10, consistent with the aforementioned macrophage phenotype changes (Figure 5A‐C), indicating ultrasound can modulate macrophages towards an anti‐inflammatory phenotype [45], but the effect is limited and insufficient for therapeutic efficacy. Treatment with free MnP or CGRP resulted in more pronounced changes in cytokine secretion compared to the ultrasound group, suggesting both ROS scavenging and CGRP alone can exert some immunomodulatory effects on macrophages. In contrast, compared to other groups, MCF@CA combined with ultrasound treatment resulted in the least secretion of pro‐inflammatory cytokines and the most secretion of the anti‐inflammatory cytokine IL‐10 (Figure 5I), consistent with the macrophage phenotype shift results. Additionally, we examined thrombospondin‐1 (TSP‐1), an extracellular matrix component secreted by macrophages, identified as a key effector molecule mediating CGRP's immunomodulatory action on macrophages, exerting anti‐inflammatory effects through autocrine and paracrine mechanisms [12]. Treatment with ultrasound, free MnP, or free CGRP increased TSP‐1 secretion by macrophages to some extent, indicating these interventions upregulated the CGRP‐RAMP‐1‐TSP‐1 signaling pathway. Compared to other groups, MCF@CA + US treatment significantly increased TSP‐1 secretion (Figure 5J), suggesting that the synergy between ROS scavenging and CGRP achieves the best neuroimmune regulation effect. Furthermore, we speculate this effect may be dose‐dependent, as the groups receiving non‐targeted nanoparticles (MC@CA+US) and MCF@CA without ultrasound‐controlled release secreted less TSP‐1 than the MCF@CA + US group, indicating that the dose of nanoparticles taken up by cells affects their biological effect. The above evidence confirms MCF@CA's immunomodulatory effect on macrophages. Notably, secretion of the pro‐angiogenic factor VEGF‐A by macrophages also increased (Figure 5G), suggesting MCF@CA might exert neuro‐immune‐vascular regulatory effects in the wound via CGRP‐mediated macrophage immunomodulation, aligning with findings from studies on healing in other tissues [46, 47, 48, 49].

### 2.5 Potential Mechanism of MCF@CA Action on Macrophages

**FIGURE 6:**
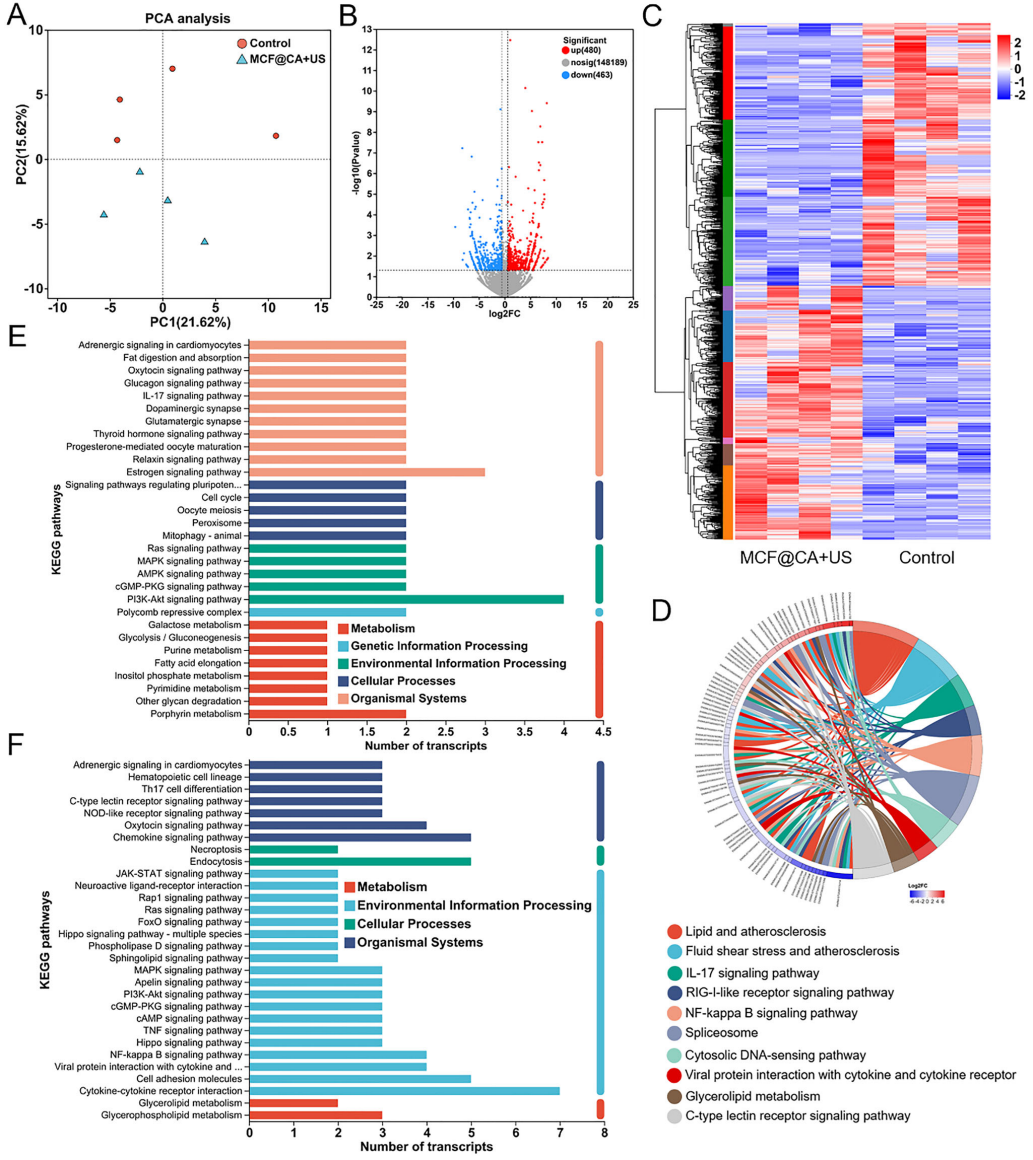
Analysis of RNA sequencing results for MCF@CA treatment. (A) PCA analysis presenting differentially expressed genes. (B) Volcano plots illustrate differentially expressed genes (gray signifies non‐significant genes; red indicates upregulated genes; blue stands for downregulated genes). (C) Heatmap representation of the DEGs. (D) A chord diagram showing KEGG enrichment terms along with their corresponding top 10 DEGs. (E) KEGG enrichment analysis presenting the upregulated genes. (F) KEGG enrichment analysis presenting the downregulated genes.

To explore the potential mechanism underlying MCF@CA's immunomodulatory effect on macrophages, we used RNA sequencing (RNA‐seq) to compare messenger RNA (mRNA) differences between MCF@CA‐treated M1 macrophages (BMDM, model constructed as in Section 2.4) and controls (Figure 6). Principal component analysis (PCA) revealed significant overall transcriptional differences between the MCF@CA and control groups (Figure 6A). A volcano plot (Figure 6B) displays the differentially expressed genes (DEGs). MCF@CA treatment resulted in 943 DEGs, including 480 upregulated and 463 downregulated genes. Figure 6C shows a heatmap of the expression patterns of these 943 genes.

Kyoto Encyclopedia of Genes and Genomes (KEGG) pathway enrichment analysis was used to interpret the cellular signaling pathways regulated by these DEGs (Figure 6D‐F). Figure 6E,F highlight the main signaling pathways associated with upregulated and downregulated genes, respectively. MCF@CA treatment upregulated genes related to the AMPK signaling pathway in macrophages. Literature reports [50] indicate AMPK activation is a key mechanism for suppressing macrophage‐mediated inflammation. We also observed upregulation of peroxisome‐related genes, potentially linked to improved oxidative stress status within macrophages. Conversely, MCF@CA downregulated the transcription of genes involved in multiple pro‐inflammatory pathways, including NOD‐like receptor, NF‐κB, TNF, and Chemokine signaling pathways. NF‐κB is recognized as a key pathway mediating immune responses in macrophages [51]. NF‐κB is essential for the transcription of NOD‐like receptor family pyrin domain containing 3 (NLRP3), which is subsequently activated by various stimuli, ultimately leading to IL‐1β release [52]. Additionally, the Chemokine signaling pathway is implicated in promoting macrophage polarization towards a pro‐inflammatory phenotype [53]. The downregulation of these pathways favors the suppression of macrophage‐mediated inflammation, further demonstrating MCF@CA's ability to exert anti‐inflammatory effects through macrophages.

Subsequently, we validated the expression changes of several key genes within these pathways using qPCR. The results showed that, compared to the PBS control group, macrophages in the MCF@CA + US treatment group exhibited significantly downregulated mRNA expression levels of NFKB1 (Figure S6A), a core pro‐inflammatory transcription factor. The expression of its key downstream effector molecules, TNF‐α (involved in the TNF pathway) and IL‐1β (involved in the NOD‐like receptor pathway), was also markedly downregulated (Figure S6B,C). Concurrently, the expression of the chemokine CCL2 was significantly reduced (Figure S6D). These results consistently demonstrate that MCF treatment effectively inhibits the activation of the NF‐κB signaling pathway in macrophages. This inhibition occurs not only at the transcriptional hub (NFKB1) [54] but also directly leads to a comprehensive reduction in the expression of downstream inflammatory mediators (TNF‐α, IL‐1β) [55, 56] and an immune cell recruitment factor (CCL2) [57]. These findings suggest, at the molecular level, the mechanism by which MCF@CA exerts its anti‐inflammatory effects: by shutting down the NF‐κB‐dependent inflammatory response program in macrophages, potentially reprogramming them into a low‐inflammatory‐response state. This conclusion is highly consistent with the RNA‐seq analysis (Figure 6) and the changes in cytokine secretion (Figure 5E,F).

### 2.6 MCF@CA Promotes Wound Healing in Diabetic Mice

**FIGURE 7:**
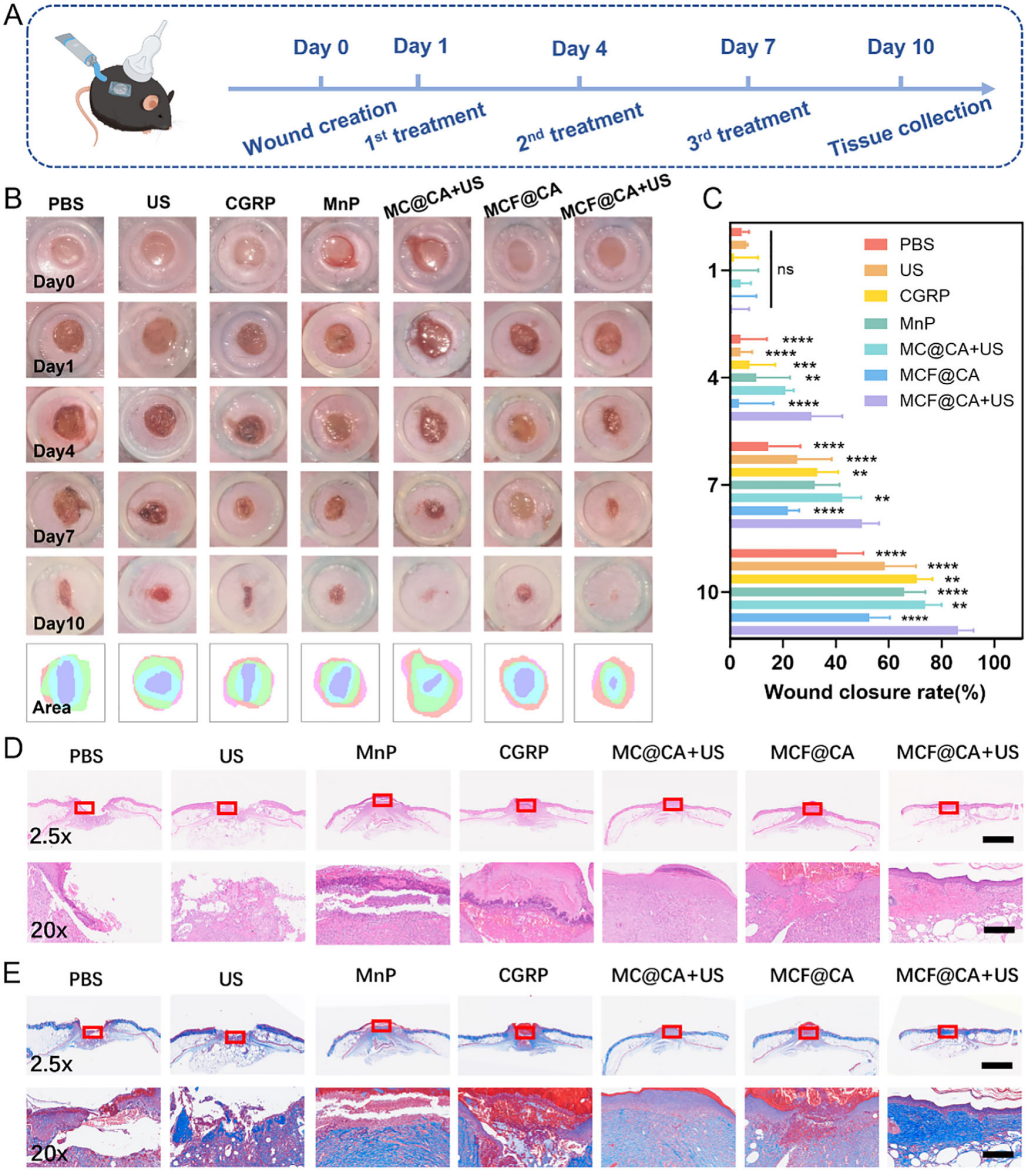
Animal Experiment Demonstrating MCF@CA Promotion of Diabetic Wound Healing. (A) Schematic of the animal experimental workflow. The picture was created by Figdraw. (B) Representative photographic images of mouse wounds under different treatments. (C) Quantitative analysis of wound closure rate across treatment groups (n = 6). Statistical significance was determined by one‐way ANOVA with intragroup comparison against the MCF@CA+US group. (D) Representative H&E‐stained sections of mouse wounds across groups. 2.5x Scale bar: 1 mm; 20x Scale bar: 100 µm. (E) Representative Masson's trichrome‐stained sections of mouse wounds across groups. 2.5x Scale bar: 1 mm; 20x Scale bar: 100 µm. Statistical significance was determined by one‐way ANOVA. *p < 0.05, **p < 0.01,***p < 0.001,****p < 0.0001.

After validating MCF@CA's ROS scavenging and immunomodulatory capabilities in vitro, we utilized diabetic mice (Lepr^db/db^) to establish a DW model to assess MCF@CA's ability to promote DW healing in vivo. Full‐thickness wounds (6 mm diameter) were created on the backs of 8‐10‐week‐old diabetic mice using a biopsy punch, and mice were randomly assigned to 7 groups. Mice received treatment on days 1, 4, and 7 post‐wounding and were sacrificed on day 10 for pathological analysis (Figure 7A). After extruding MCF@CA hydrogel from a syringe to cover the wound, an ultrasound physiotherapy device was used to trigger hydrogel degradation. Within the first 4 days post‐wounding, groups other than MC@CA and MCF@CA + US showed no significant trend of promoting wound healing (Figure 7B,C). This indicates that CGRP‐mediated neuroimmune modulation combined with ROS scavenging has a stronger pro‐healing effect than using free CGRP or ROS scavenging alone, validating the aforementioned inference about their synergistic mechanism. In the later observation period, although wounds in other groups healed to varying degrees, the MCF@CA + US group exhibited a higher wound closure rate, reaching twice that of the PBS group, and some wounds showed complete closure (Figure 7B). Furthermore, MCF@CA demonstrated better therapeutic efficacy than hydrogel loaded with non‐targeted nanoparticles (MC@CA) (Figure 7B,C), validating our previous inference that FA targeting enhances cellular uptake and prolongs nanoparticle retention in the wound, thereby improving efficacy. Another interesting finding was that the group receiving MCF@CA hydrogel *without* ultrasound application had a significantly lower wound closure rate than the group *with* ultrasound‐triggered release (Figure 7B,C), confirming ultrasound as the key factor controlling nanoparticle release.

**FIGURE 8:**
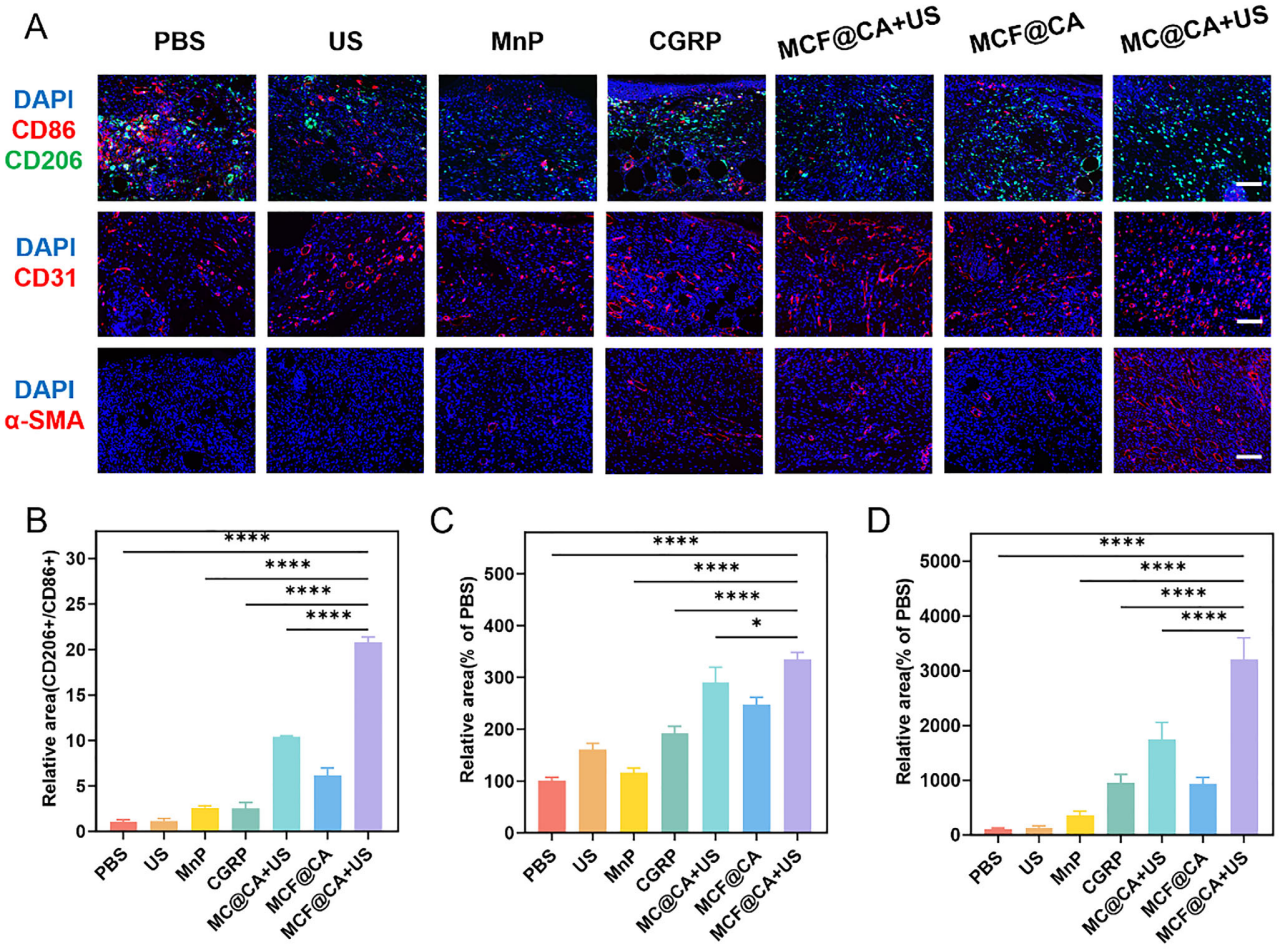
Immunofluorescence Staining Elucidating the Mechanism of MCF@CA in Promoting Wound Healing. (A) Immunofluorescence staining images of CD31, α‐SMA, and CD86(red)+CD206(green) in diabetic mouse wounds across treatment groups. Scale bar: 100 µm. (B) Quantitative analysis of CD31‐stained fluorescent area (n = 3). Statistical significance was determined by one‐way ANOVA. (C) Quantitative analysis of α‐SMA‐stained fluorescent area (n = 3). Statistical significance was determined by one‐way ANOVA. (D) Quantitative analysis of CD206/CD86 fluorescence area ratio (n = 3). Statistical significance was determined by one‐way ANOVA. *p < 0.05, **p < 0.01, ***p < 0.001, ****p < 0.0001.

Subsequently, we used histochemical staining and immunofluorescence to investigate the mechanisms of MCF@CA‐enhanced DW healing (Figures 7D,E and 8). H&E staining of wound cross‐sections showed that wounds treated with PBS or ultrasound alone remained completely open, exposing deep connective tissue. Wounds treated with free CGRP, MnP, or MCF@CA without ultrasound showed varying degrees of granulation tissue coverage but were not fully closed. In contrast, wounds treated with MC@CA or MCF@CA combined with ultrasound were completely healed, with the MCF@CA + US group showing intact skin coverage (Figure 7D; Figure S7). Masson's trichrome staining assessed collagen deposition (Figure 7E; Figure S8), a critical factor in skin regeneration. Evaluation was impossible for PBS and ultrasound alone groups due to complete wound rupture. Compared to other groups, the MCF@CA + US group showed significantly more collagen deposition after healing, beneficial for repair.

Subsequently, immunofluorescence further explored immune modulation and angiogenesis during healing. We used red fluorescence to label the M1 macrophage surface marker CD86 and green fluorescence to label the M2 macrophage surface marker CD206. The ratio of CD206+ to CD86+ cells was quantified to assess the wound's immune status (Figure 8A,B; Figure S9). Wounds treated with PBS, ultrasound, free CGRP, or free MnP contained abundant pro‐inflammatory M1 macrophages. In contrast, wounds treated with MC@CA or MCF@CA showed a reversal in macrophage phenotype within granulation tissue, predominantly exhibiting the anti‐inflammatory M2 phenotype. This indicates that CGRP synergizing with ROS scavenging achieves better neuroimmune regulation. Notably, compared to the group with non‐targeted nanoparticles (MC@CA), the MCF@CA + US group had a higher proportion of M2 macrophages, further proving that FA targeting prolonging MCF NP retention in the wound benefits healing. Next, we used immunofluorescence to label vascular markers CD31 and α‐SMA to investigate the effect of MCF@CA on wound angiogenesis. Expression of the vascular endothelial marker CD31 significantly increased after MCF@CA + US treatment, with the fluorescent area increasing to over 300% of the PBS group, and distinct tubular structures were observed (Figure 8A,C; Figure S10), indicating enhanced neovascularization in granulation tissue. This aligns with the trend of increased pro‐angiogenic factor VEGF‐A secretion by macrophages observed in the aforementioned in vitro experiment (Figure 5J), demonstrating that MCF@CA enhances macrophage‐mediated pro‐angiogenesis through immunomodulation. Notably, staining for α‐SMA (marking vascular smooth muscle cells) revealed numerous vessels with smooth muscle coverage in the MCF@CA + US group, exceeding 30 times that of the PBS group (Figure 8A,D; Figure S11). This indicates that MCF@CA not only increases capillary density in diabetic mouse wounds but also promotes the formation of larger vessels with more advanced structures, such as arterioles or venules, which is beneficial for further reversing the hypo vascular state of DW.

In summary, MCF@CA combined with ultrasound effectively promoted DW healing in the animal model. Specifically, it enhanced wound closure rate and increased collagen deposition. Immunofluorescence confirmed MCF@CA promotes macrophage polarization towards an anti‐inflammatory phenotype while increasing vascular density and promoting the formation of larger, smooth muscle‐covered vessels. These findings align with our in vitro observations of improved fibroblast function, macrophage phenotype modulation, and alterations in inflammatory and pro‐angiogenic cytokines, further corroborating MCF@CA's mechanism of promoting DW healing through ROS scavenging and immunomodulation.

### 2.7 Biocompatibility Evaluation of MCF@CA

**FIGURE 9:**
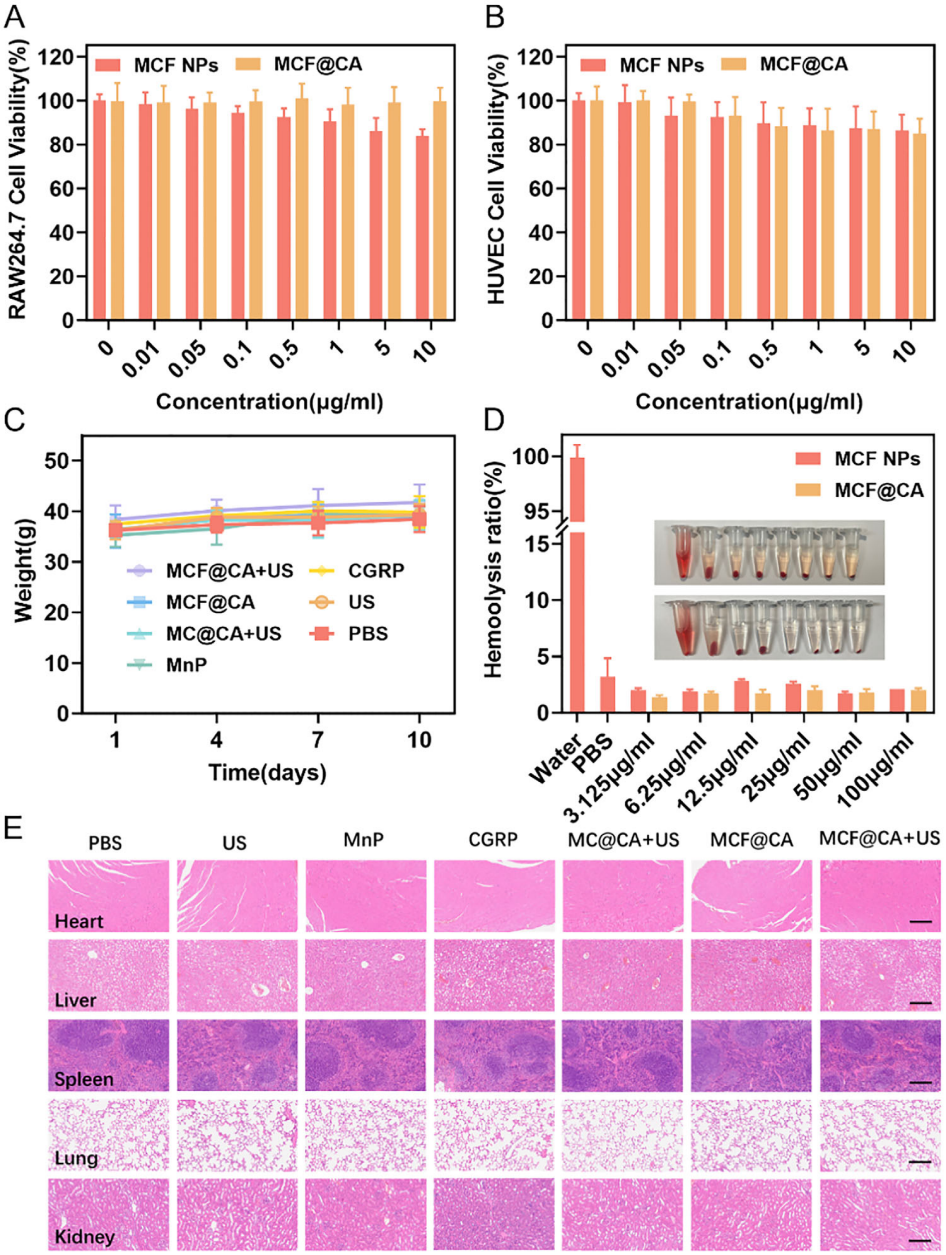
Biocompatibility Evaluation of MCF NPs and MCF@CA. (A) Cell viability of RAW 264.7 cells treated with different concentrations of MCF NPs and MCF@CA for 24 h, measured by CCK‐8 assay (n = 3). (B) Cell viability of HUVEC cells treated with different concentrations of MCF NPs and MCF@CA for 24 h, measured by CCK‐8 assay (n = 3). (C) Body weight changes of mice in different treatment groups during the therapeutic period. (D) Hemolysis assay of MCF NPs and MCF@CA (n = 3). (E) H&E‐stained sections of major organs from mice in different treatment groups after therapy completion. Scale bar: 200 µm.

Wound dressings require sufficient safety for clinical translation. Therefore, we evaluated the biocompatibility of MCF@CA in vitro and in vivo. First, cytotoxicity of MCF NPs and MCF@CA was assessed using RAW264.7 cells and human umbilical vein endothelial cells (HUVECs) (Figure 9A,B). The CCK‐8 assay showed that HUVEC viability did not decrease significantly with increasing drug concentration. Even at 10 µg/mL, cell viability remained above 80% for both MCF NPs and MCF@CA, a concentration far exceeding the effective therapeutic concentration of MCF NPs (100 ng/mL), indicating good biocompatibility. We then verified MCF@CA's safety in diabetic mice. Body weight monitoring showed no significant differences among groups during treatment (Figure 9C). Hemolysis assays confirmed no significant hemolysis caused by MCF NPs or MCF@CA at 100 µg/mL (Figure 9D). Histopathological examination (H&E staining) of major organs post‐treatment revealed intracellular fat vacuoles in the livers of all groups. This is attributed to spontaneous fatty liver, a consequence of the inherent metabolic abnormalities in diabetic mice. Apart from this, the histological structure of visceral organs in the treatment groups showed no other abnormalities (Figure 9E). These experiments demonstrate the good biocompatibility of MCF@CA, laying the foundation for future clinical translation.

## 3 Conclusions

The treatment of DW is a slow process requiring low‐dose, long‐term drug action. Therefore, a platform capable of controlled drug release on demand is essential. Furthermore, current treatments neglect the important upstream regulatory mechanism of neuroimmune communication. In this study, we constructed the MCF@CA hydrogel, demonstrating excellent capability in promoting DW healing. MCF@CA allows controlled drug release rate by ultrasound‐triggered disintegration of the calcium‐crosslinked network, enabling future adjustment based on diverse clinical needs. Through ROS scavenging and CGRP‐mediated neuroimmune modulation, MCF@CA effectively ameliorates intracellular oxidative stress, improves fibroblast proliferation and migration, and promotes macrophage polarization towards the anti‐inflammatory M2 phenotype. Experiments in a diabetic mouse wound model demonstrated that MCF@CA accelerates DW healing by modulating wound macrophage phenotype and promoting the formation of larger, smooth muscle‐covered blood vessels. Overall, the MCF@CA hydrogel shows significant promise for developing novel therapeutics for DW treatment.

## 4 Experimental Section

### 4.1 Materials

5‐(4‐Carboxyphenyl)‐10,15,20‐triphenylporphyrin was purchased from Aladdin (Shanghai, China). Calcitonin gene related peptide (CGRP) was purchased from Tanshtech Co., Ltd (GuangZhou, China). Cell counting kit‐8 was purchased from LABLEAD CO., Ltd. (Beijing, China). 2′,7′‐Dichlorofluorescein diacetate (DCFH‐DA) was purchased from Sigma‐Aldrich (Shanghai, China). The PE anti‐mouse CD206, Brilliant Violet 605 anti‐mouse CD86, APC anti‐mouse CD86 were purchased from Biolegend (USA). RAMP1 Polyclonal antibody was purchased from Proteintech Group, Inc (Hubei, China).

### 4.2 Cell Culture

Mouse Monocytic Macrophage Leukemia Cell Line (RAW 264.7 cells, RRID: CVCL_UL71) and rat skin fibroblast‐like cells (RS1 cells, RRID: CVCL_DR20) were purchased from Pricella Biotechnology Co., Ltd. (China, Wuhan). RAW 264.7 cells were cultured in high‐glucose Dulbecco's modified Eagle's medium (DMEM), supplemented with fetal bovine serum (FBS, 10% (v/v)), 1% penicillin‐streptomycin (v/v). RS1 cells were cultured in RPMI‐1640 medium, supplemented with fetal bovine serum (FBS, 15% (v/v)), 1% penicillin‐streptomycin (v/v). Mouse bone marrow‐derived macrophages (BMDMs) were isolated from the femurs and tibias of 6‐week‐old female C57BL/6N mice. Cells were cultured in DMEM supplemented with 10% FBS (v/v), 1% penicillin‐streptomycin (v/v), and 20 ng/mL macrophage colony‐stimulating factor (M‐CSF) for 5 days to induce differentiation into macrophages. The cells were cultured in an incubator with 5% CO_2_ at 37°C. All cell lines used in this study were regularly tested and confirmed to be free of mycoplasma contamination.

### 4.3 Preparation of MCF NPs

18.4 µmol of 5‐(4‐Carboxyphenyl)‐10,15,20‐triphenylporphyrin, 105 µmol manganese chloride (MnCl_2_), and 432 µmol sodium acetate were dissolved in 5 mL glacial acetic acid. The mixture was stirred at 60°C for 2 h in an oil bath to synthesize MnP. The resulting mixture was subjected to ethanol injection to aggregate MnP, followed by dialysis purification and lyophilization to obtain purified MnP. MnP and 1‐ethyl‐3‐(3‐dimethylaminopropyl) carbodiimide (EDC) at a 1:2 molar ratio were dissolved in N,N‐dimethylformamide (DMF) and stirred at room temperature for 10 min. N‐hydroxysuccinimide (NHS) (1.2 equivalents relative to MnP, n/n) was added, and the mixture was stirred at room temperature for 1 h. Subsequently, CGRP (1.2 equivalents relative to MnP, n/n) was added, and the reaction proceeded overnight to yield MnP‐CGRP (MC).

MCF NPs were prepared using the ethanol injection method. MC and DSPE‐PEG2K‐FA were co‐dissolved in DMSO (10:1 molar ratio, n/n), with the MC concentration at 10 mg/mL. The solution was injected into ddH_2_O (1:7, v/v) under sonication in a water bath. The mixture was then dialyzed extensively against ddH_2_O and lyophilized to obtain MCF NPs powder.

The hydrodynamic diameter of nanoparticles in solution was measured by dynamic light scattering (DLS), while the particle size under dry state was determined by transmission electron microscopy (TEM).

### 4.4 Preparation of MCF@CA

300 mg sodium alginate and 0.1 mg MCF NPs were dissolved in 8 mL ddH_2_O. 84 mg CaSO_4_ powder was dispersed in 2 mL ddH_2_O to form a suspension. The two solutions/suspensions were then rapidly mixed using syringes to form the MCF@CA hydrogel. To simulate the in vivo process of hydrogel degradation under ultrasound and sustained MCF NP release into the wound for in vitro experiments, while avoiding Transwell chamber obstruction of ultrasound, MCF@CA was pre‐immersed in culture medium (3:100, v/v) and irradiated with an ultrasound physiotherapy device (20% duty cycle, 0.3 W/cm^2^, 1 MHz) for 24 h. The drug‐containing medium was then collected, filtered sterilized, and used for cell treatment.

### 4.5 Fluorescence Imaging of Intracellular ROS Levels

RAW 264.7 cells and RS1 cells were seeded in 12‐well cell culture plates and cultured at 37°C for 24 h. The cells were incubated with H_2_O_2_ (100 nM) for 4 h, then incubated with medium treated by MCF@CA, followed by incubation with 10 µM DCFH‐DA for 30 min. After the cells were washed with PBS two times, the images were obtained by fluorescence microscope with an excitation wavelength of 488 nm.

### 4.6 In Vitro Macrophage Phenotype Modulation Assay

Mouse bone marrow‐derived macrophages (BMDMs) were isolated from the femurs and tibias of 6‐week‐old female C57BL/6N mice. Cells were cultured in DMEM supplemented with 10% FBS, 1% penicillin‐streptomycin, and 20 ng/mL macrophage colony‐stimulating factor (M‐CSF) for 5 days to induce differentiation into macrophages. For M1 polarization, cells were treated with 100 ng/mL lipopolysaccharide (LPS) for 24 h. For M2 polarization (control), cells were treated with 50 ng/mL interleukin‐4 (IL‐4) for 48 h. Subsequently, cells were incubated with MCF@CA‐conditioned medium (prepared as in 4.4) for 48 h before analysis.

### 4.7 RNA Extraction, cDNA Synthesis, and Quantitative Real‐Time PCR (qPCR)

Total RNA was extracted from BMDMs using TRIzol reagent according to the manufacturer‘s protocol. Briefly, cells were lysed in TRIzol, followed by the addition of chloroform and phase separation by centrifugation. The aqueous phase was transferred, and RNA was precipitated with isopropanol at ‐20°C overnight. The RNA pellet was washed with 75% ethanol, air‐dried, and finally dissolved in RNase‐free water. RNA concentration and purity were assessed by spectrophotometry (NanoDrop). To eliminate genomic DNA contamination, 1 µg of total RNA was treated with RNase‐free DNase I. First‐strand cDNA was then synthesized from 2 µg of DNase‐treated RNA using the Mighty Script Plus First Strand cDNA Synthesis Master Mix.

Quantitative real‐time PCR (qPCR) was performed using a CFX96 Real‐Time PCR Detection System. Each 15 µL reaction contained the equivalent of 3 ng of initial RNA, 200 nM of each gene‐specific primer (sequences listed in Table S3), and the 2× SG Fast qPCR Master Mix (B639272, Sangon Biotech). The thermal cycling protocol was as follows: initial denaturation at 95°C for 10 min; 40 cycles of 95°C for 30 s, 65°C for 30 s (with fluorescence acquisition), and 72°C for 30 s; followed by a dissociation curve analysis to verify amplification specificity. The expression level of each target gene was normalized to the endogenous control β‐actin, and the relative fold change was calculated using the 2^(‐ΔΔCt) method.

Primers were as follows (5'–3'): NFKB1, F: TAGAATTGCCCCTACCCAGC, R: AGGAGCAGGACATGGGATTT; TNF, F: TCTTCTCATTCCTGCTTGTGG, R: GATCTGAGTGTGAGGGTCTGG; IL1B, F: GCAACTGTGTTCCTGAACTCAACT, R: ATCTTTTGGGGGTCCGTCAACT; CCL2, F: CACTCACCTGCTGCTACTCA, R: GCTTGGTGACAAAAACTACAGC.

### 4.8 Animals and Wound Model Establishment

Lepr^db/db^ mice (10 weeks old, male) were purchased from Gem Pharmatech Co., Ltd. All animal experiments were performed in accordance with the guidelines of the Peking University Laboratory Animal Management Committee. Full‐thickness excisional wounds (6 mm diameter) were created on the dorsal skin using a sterile biopsy punch. Silicone rings (10 mm inner diameter) were fixed around the wounds using tissue adhesive to prevent wound contraction. The study protocol was approved by the Institutional Animal Care and Use Committee of Peking University Health Science Center (DLASBE0192).

### 4.9 Statistical Analysis

Data were expressed as mean ± standard deviation (SD). The sample size (n) for each experiment, representing the number of independent biological replicates, is provided in the corresponding figure legend. For comparisons between two groups of normally distributed data, two‐tailed, unpaired Student's *t*‐test was performed. For comparisons among more than two groups, ordinary one‐way analysis of variance (ANOVA) was applied. The threshold for statistical significance was set at a *p* value of less than 0.05. Asterisks indicated significant differences (*p < 0.05, **p < 0.01, ***p < 0.001, ****p < 0.0001). All statistical analyses were performed using GraphPad Prism software (version 10.1.2).

## Conflicts of Interest

The authors declare no conflict of interest.

## Supporting information




**Supporting File**: advs73920‐sup‐0001‐SuppMat.docx.

## Data Availability

The data that support the findings of this study are available from the corresponding author upon reasonable request.
