# Supplementary material for: Ultrasound Controlled‐Release Hydrogel Promotes Diabetic Wound Healing via Neuroimmune Modulation and Synergistic ROS Scavenging
Source: Adv Sci (Weinh). 2026 Jan 21;13(18):e16882. doi: 10.1002/advs.202516882 (PMC13042872; doi:10.1002/advs.202516882)
Supplement: Supplementary file 1 — Supporting File: advs73920‐sup‐0001‐SuppMat.docx. [file ADVS-13-e16882-s001.docx]

Supporting Information

**Ultrasound Controlled-Release Hydrogel Promotes Diabetic Wound Healing via Neuroimmune Modulation and Synergistic ROS Scavenging**

*Mofan Li^#^, Mengxin Wang^#^, Haonan Wang, Yang Sun, Yongyue Zhang, Shuyu Xu, Tianjiao Zhang, Shiti Shama, Xiaolong Liang*, Shumin Wang**

Department of Ultrasound, Peking University Third Hospital, Beijing 100191, China.

*^#^ Mofan Li and Mengxin Wang contributed equally to this work.*

* Corresponding author:

*Xiaolong Liang*

*xiaolong_liang@bjmu.edu.cn*

*Shumin Wang*

shuminwang@bjmu.edu.cn

**（A）**


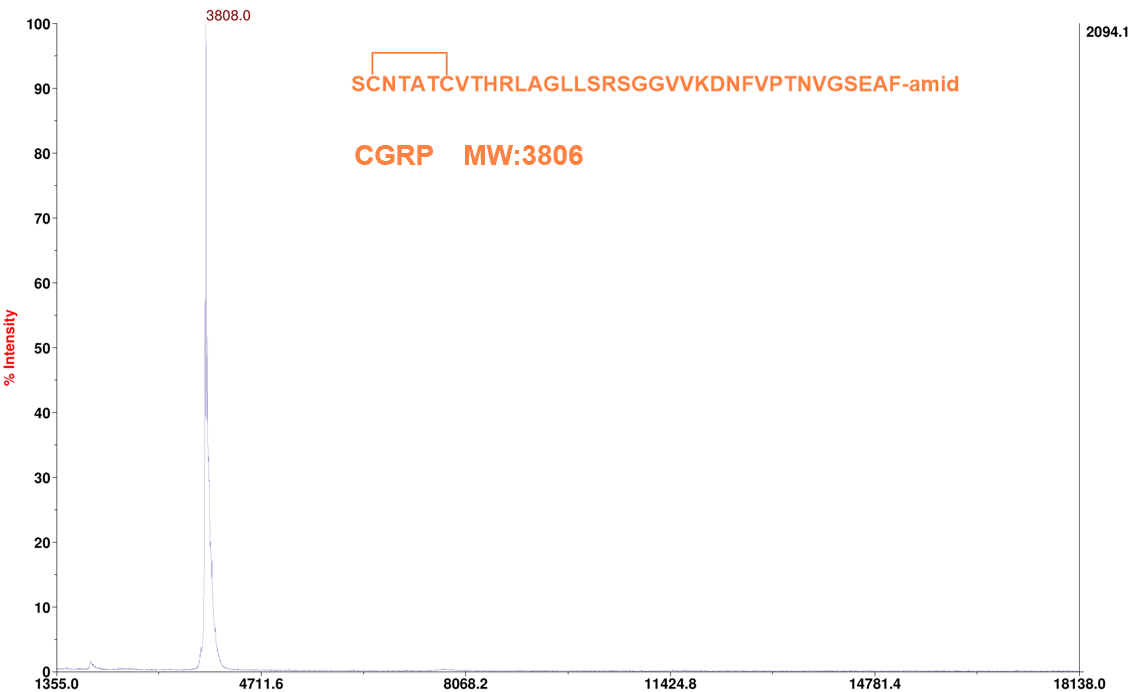


**（B）**


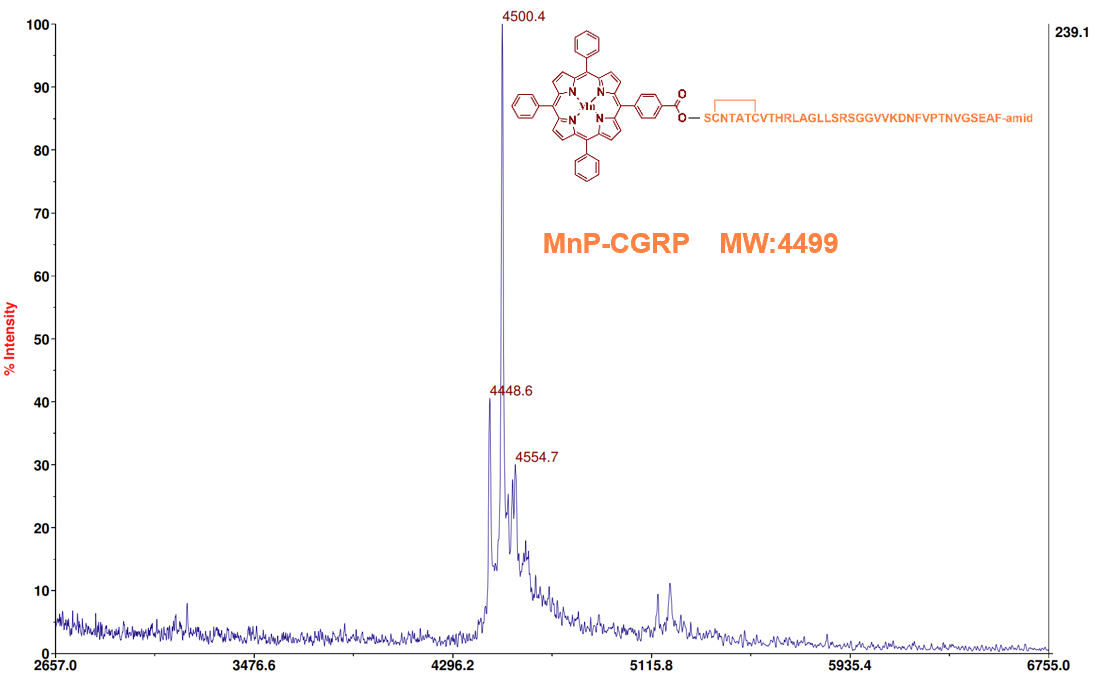


**Figure S1. Mass spectrometry characterization of CGRP (A) and MnP-CGRP (B)**


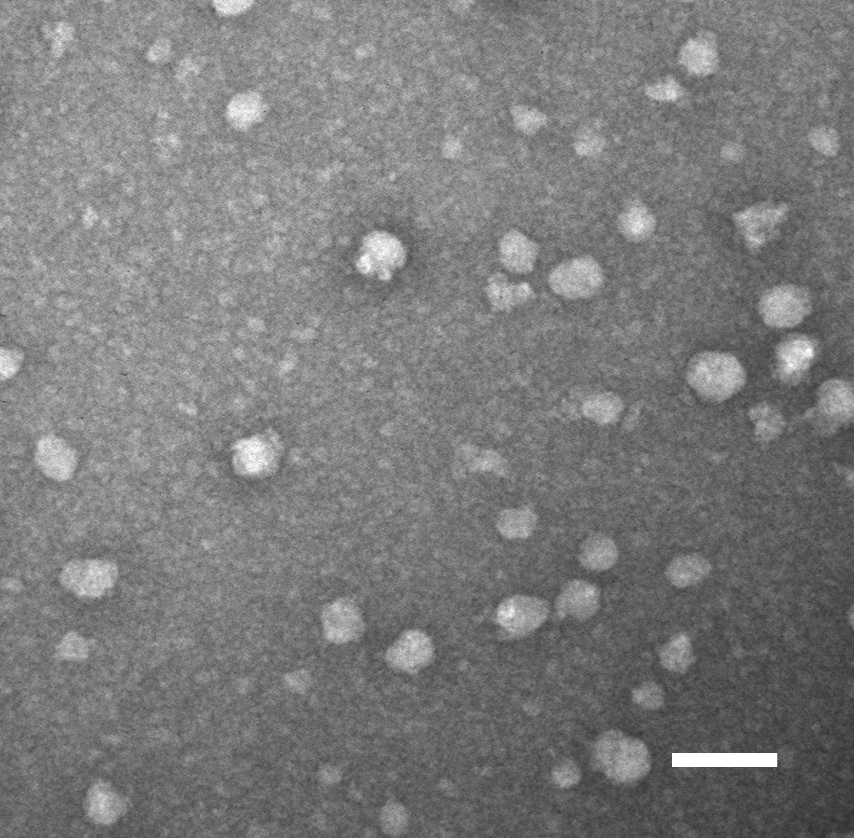


**Figure S2. High-resolution version of TEM image in Figure 2D. Scale bar: 100 nm.**

**（A）**


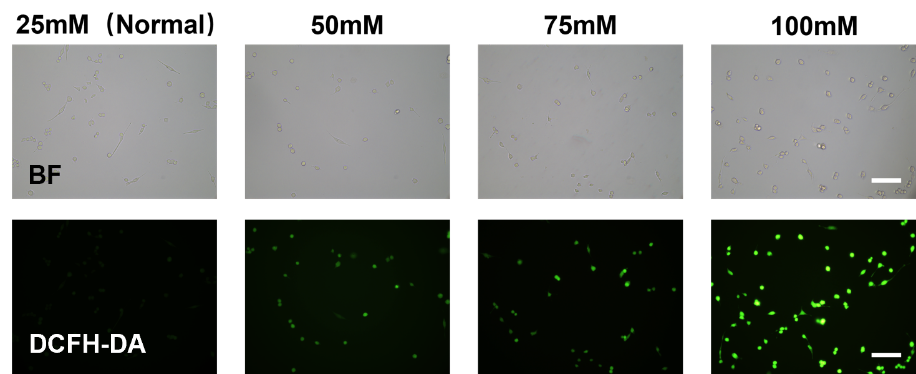


**（B）**

**Figure S3. Fluorescence images of intracellular ROS in RAW 264.7 cells treated with different glucose concentrations (A) and quantitative analysis of DCFH-DA Fluorescent area (B) (n=3). Scale bar: 500 μm.**

**（A）**


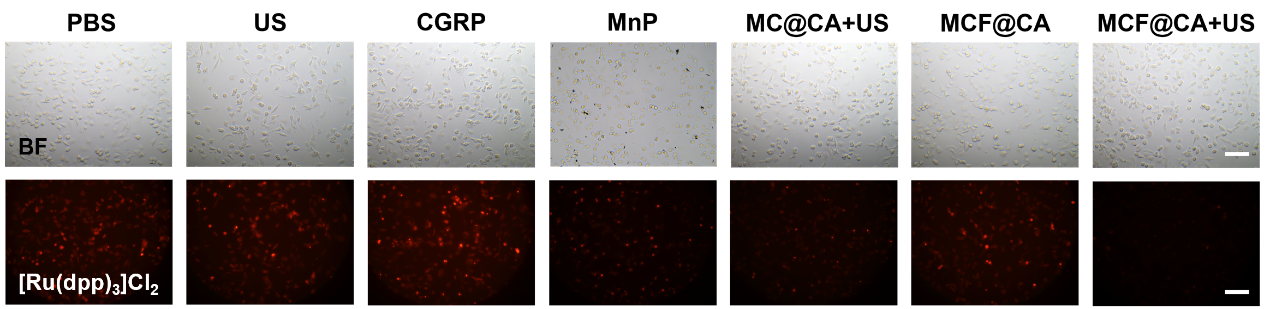


**（B）**

**Figure S4. Fluorescence imaging of hypoxia status in RAW 264.7 cells under different treatments (A) and quantitative analysis of [Ru(dpp)_3_]Cl_2_ fluorescent area (B) (n=3). Scale bar: 500μm.**

**Figure S5. Impact of different hydrogen peroxide concentrations on RS1 cell proliferation measured by CCK-8 assay (n=3).**


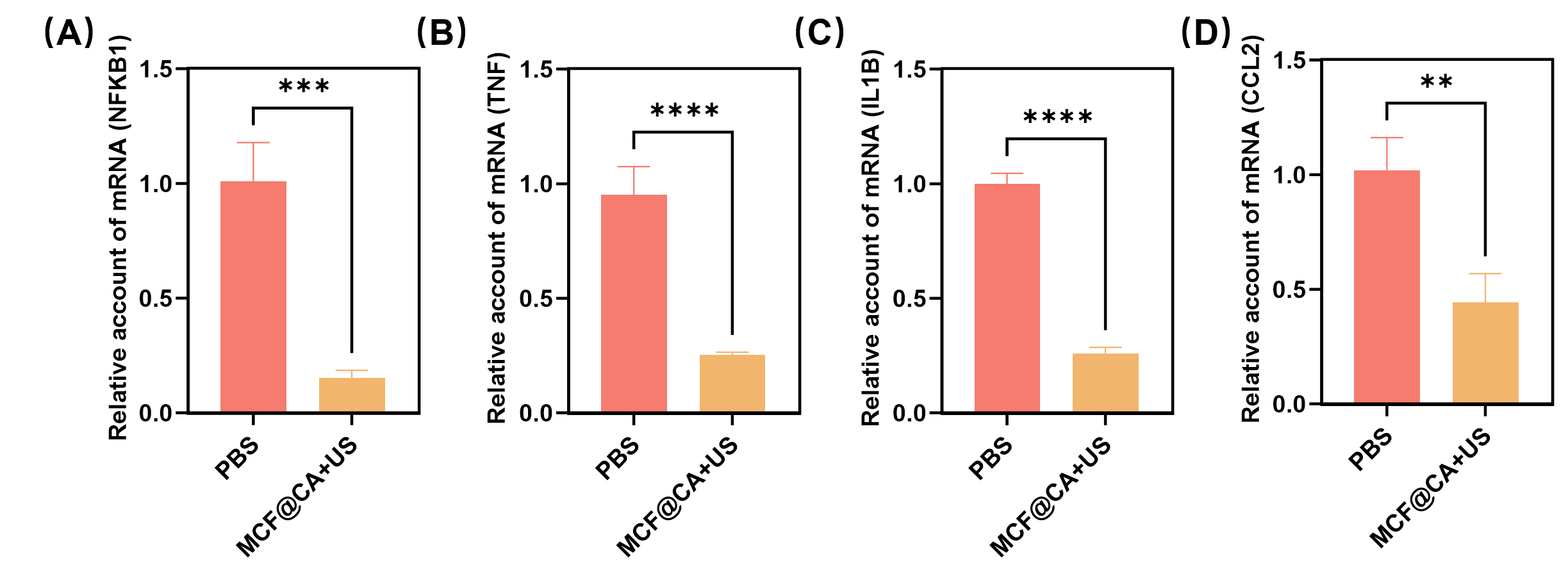


**Figure S6. Changes in mRNA levels of inflammation-related genes in M1-type macrophages were detected by qPCR (n=3)**. A. NFKB1. B. TNF. C. IL1B. D. CCL2. Statistical significance was determined by student t-test. *p<0.05, **p<0.01,***p<0.001,****p<0.0001

**
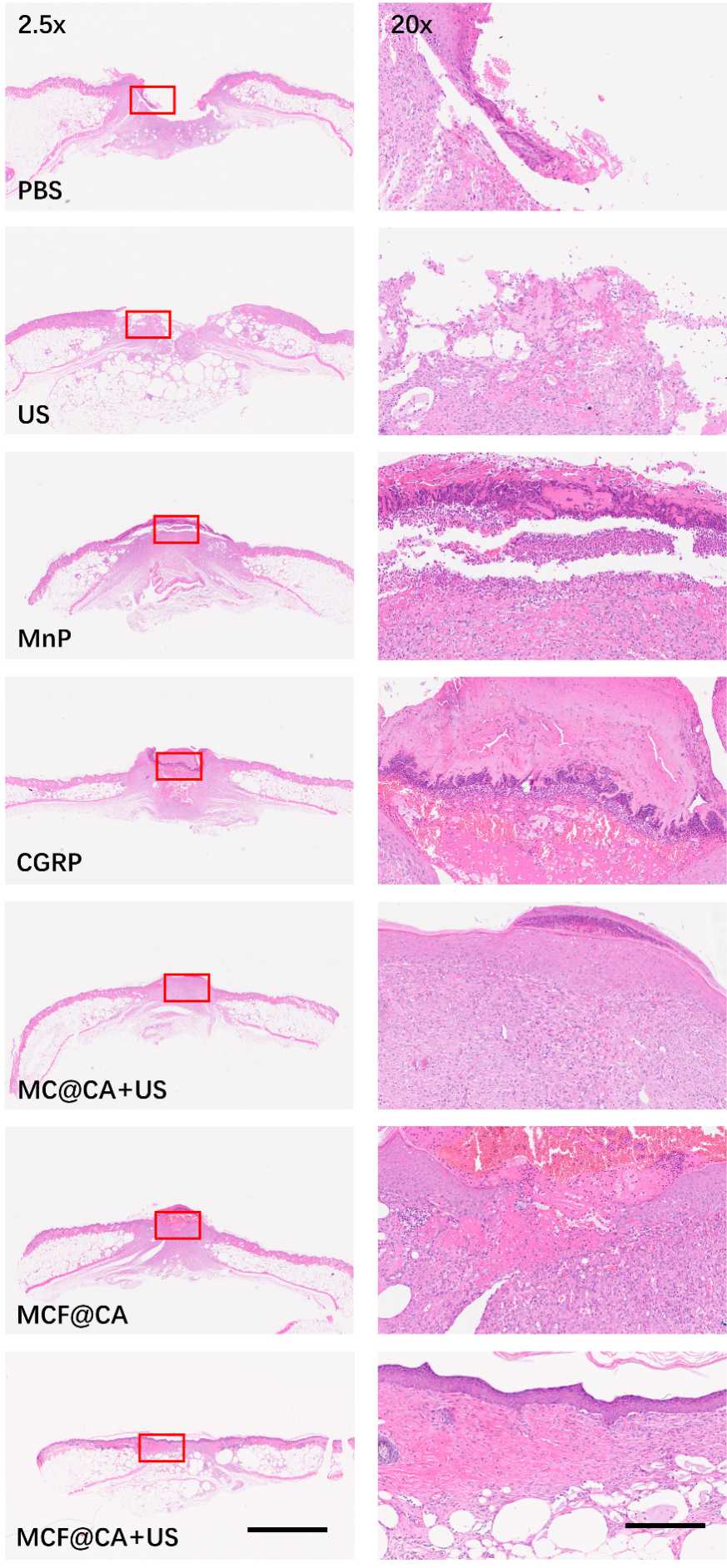
**

**Figure S7. High-resolution version of Figure 7D. 2.5x Scale bar：1mm；20x Scale bar：100μm.**

**
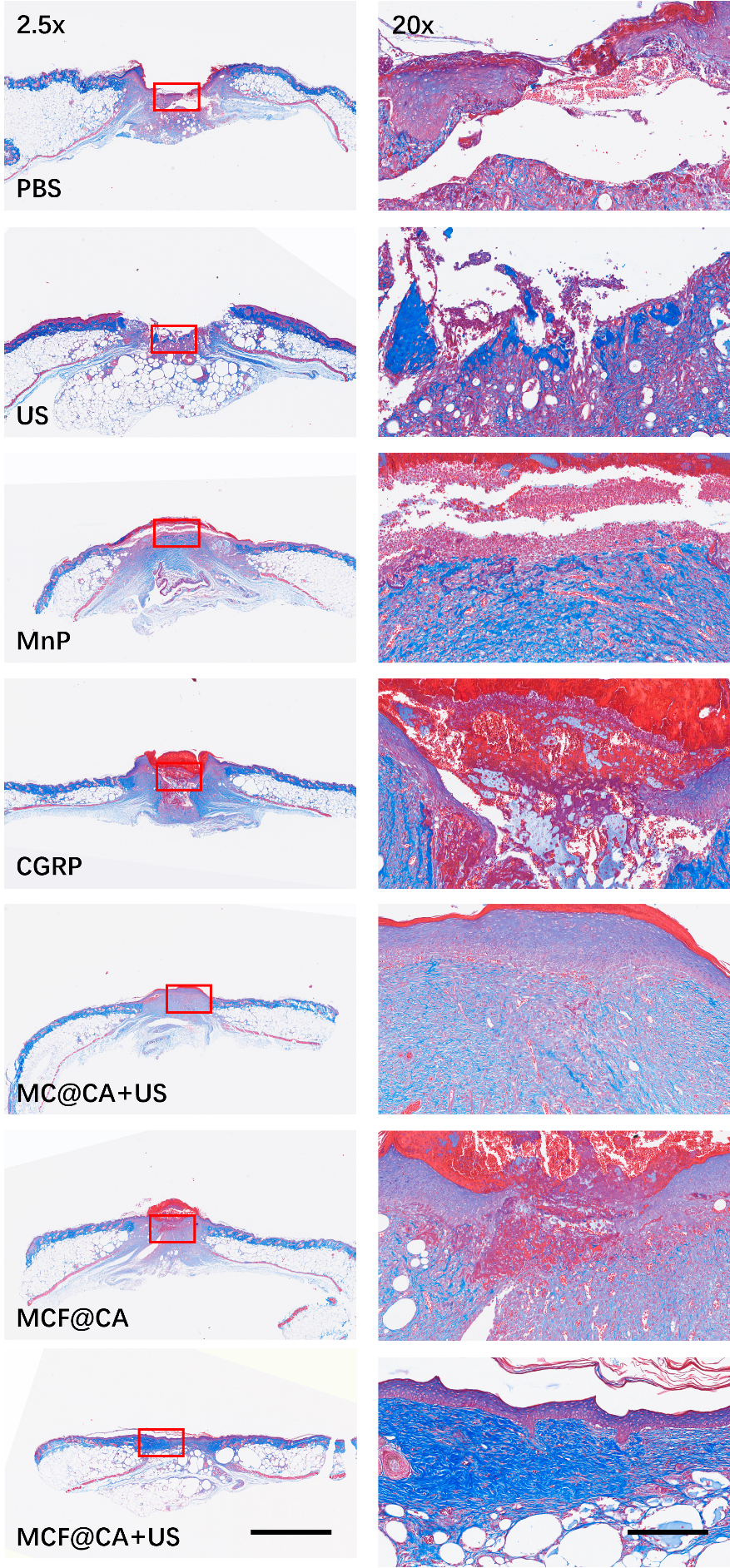
**

**Figure S8. High-resolution version of Figure 7E. 2.5x Scale bar：1mm；20x Scale bar：100μm.**


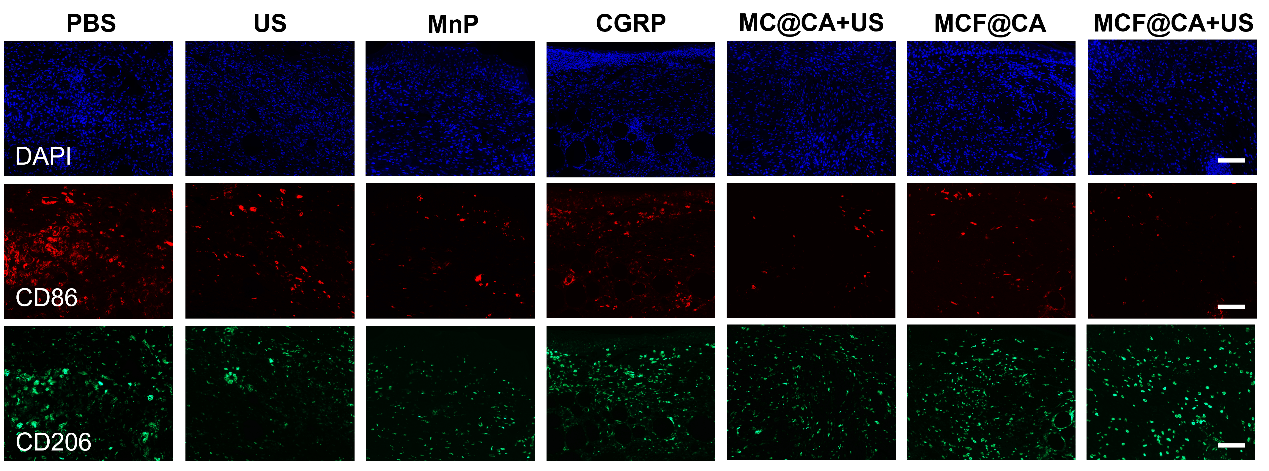


**Figure S9. Immunofluorescence staining images of CD86(red)+CD206(green) in diabetic mouse wounds across different groups. Scale bar: 100μm.**


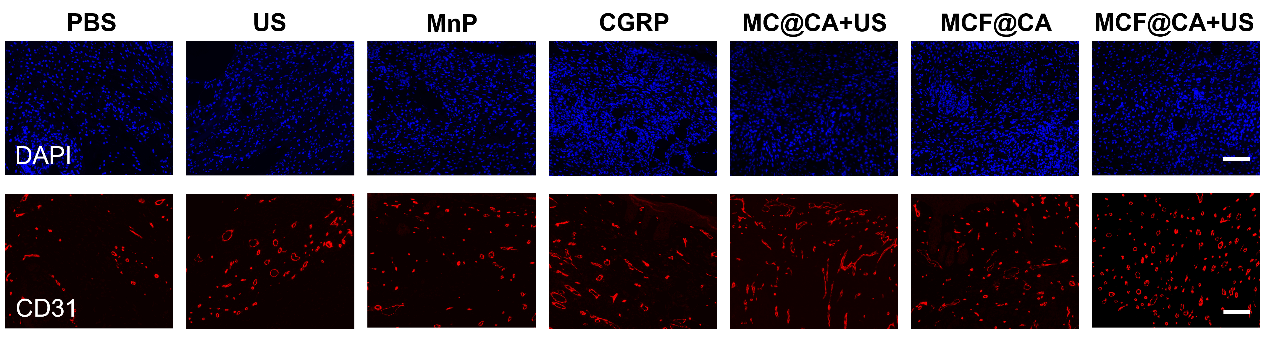


**Figure S10. Immunofluorescence staining images of CD31 in diabetic mouse wounds across different groups. Scale bar: 100μm.**

**
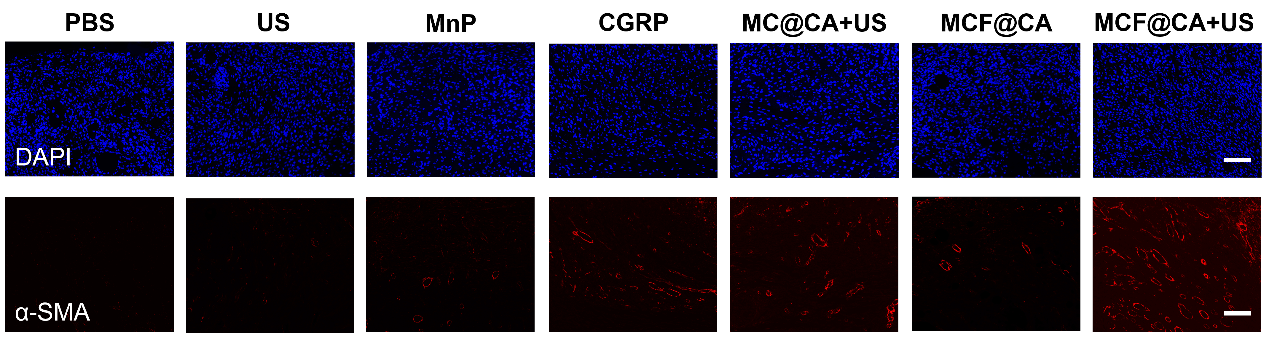
**

**Figure S11 Immunofluorescence staining images of α-SMA in diabetic mouse wounds across different groups. Scale bar: 100μm.**
